# Integrated Chemical, Computational, and Cellular Profiling of a Dual-Oil Melanoma Formulation: An Exploratory Life-Science Study

**DOI:** 10.3390/life16081314

**Published:** 2026-08-11

**Authors:** Katarina Dunjic, Momir Dunjic, Marina Gazdic Jankovic, Marina Miletic Kovacevic, Nikolina Kastratovic, Biljana Ljujic, Tatjana Novakovic, Milan Filipovic, Tatjana Filipovic, Zhao Jing, Marija Dunjic, Miroslav M. Sovrlić, Sandra S. Konstantinović, Jelena S. Stanojević, Milan D. Kostić, Stefano Turini

**Affiliations:** 1 Discipline of Dermato-Venereology, Quince Medic Clinic, Bulevar Oslobodjenja 2, 11000 Belgrade, Serbia; dunjickatarina@gmail.com; 2 Discipline of Obstetrics/Gynecology and Integrative Medicine, Faculty of Medicine, University of Pristina, Anri Dinana bb, 38220 Kosovska Mitrovica, Serbia; 3 Faculty of Pharmacy, Heroja Pinkija 4, 21000 Novi Sad, Serbia; 4 Department of Physical Medicine, Alma Mater Europaea (AMEU-ECM), Slovenska Ulica 17, 2000 Maribor, Slovenia; turini.stefano@yahoo.it; 5 Department of Genetics, Faculty of Medical Sciences, University of Kragujevac, 34000 Kragujevac, Serbian_kastratovic@outlook.com (N.K.); bljujic74@gmail.com (B.L.); 6 Department of Histology and Embryology, Faculty of Medical Sciences, University of Kragujevac, 34000 Kragujevac, Serbia; marina84kv@gmail.com; 7 Discipline of Internal Medicine, Faculty of Medicine, University of Pristina, Anri Dinana bb, 38220 Kosovska Mitrovica, Serbia; novakovictanja65@gmail.com; 8 Surgery Department, Discipline of Plastic and Reconstructive Surgery, Faculty of Medicine, University of Pristina, Anri Dinana bb, 38220 Kosovska Mitrovica, Serbia; milan.filipovic1967@gmail.com; 9 Discipline of Anatomy, Faculty of Medicine, University of Pristina, Anri Dinana bb, 38220 Kosovska Mitrovica, Serbia; tatjana.filipovic1967@gmail.com; 10 Institute of Basic Research in Clinical Medicine, China Academy of Chinese Medical Sciences (CACMS), Dongcheng District, Beijing 100700, China; hhzhaojing@hotmail.com; 11 Discipline of Plastic and Reconstructive Surgery, Quince Medic Clinic, Bulevar Oslobodjenja 2, 11000 Belgrade, Serbia; marijadunjic@yahoo.com; 12 Department of Pharmacy, Faculty of Medical Sciences, University of Kragujevac, 34000 Kragujevac, Serbia; sofke-ph@hotmail.com; 13 Faculty of Technology, University of Niš, 16000 Leskovac, Serbia; sakisandra12@yahoo.com (S.S.K.); jstanojevic@tf.ni.ac.rs (J.S.S.); milan@tf.ni.ac.rs (M.D.K.); 14 Division of Advanced Research and Development, Worldwide Consultancy and Services, Via Andrea Ferrara 45, 00165 Rome, Italy; 15 Division of Environmental Medicine and Security, Capri Campus Forensic and Security, Via G. Orlandi 91, 80071 Anacapri, Italy; 16 Pancic Institute for Research on Medicinal Plants, Institute of Medical Research, University of Belgrade, Dr Subotića 4, 11029 Belgrade, Serbia

**Keywords:** malignant melanoma, *Prunus dulcis*, *Pinus sylvestris*, α-pinene, dual-oil formulation, GC-MS, FAME, molecular docking, three-dimensional docking complexes, neutral red uptake assay, B16F10, MRC-5, exact Spearman correlation, exploratory life-science study

## Abstract

Background: Previous studies have examined bioactive constituents of *Prunus dulcis* oil, *Pinus sylvestris* essential oil, and related phytochemical matrices separately; however, their combined chemical profile, comparator-context molecular-docking landscape, and comparative cellular response in melanoma and non-malignant fibroblast models remain insufficiently characterized. Methods: The analyzed study samples were profiled by GC/MS and GC/FID-based fatty-acid methyl ester analysis. An archived molecular-docking dataset was used to summarize structurally plausible interactions of six formulation-associated markers with eight melanoma-relevant or melanoma-adjacent targets and to present representative three-dimensional complexes. Because complete protocol metadata and co-crystallized-ligand redocking records were unavailable, the computational findings were interpreted descriptively. Neutral-red uptake assays assessed the 24 h viability of B16F10 melanoma cells and MRC-5 fibroblasts after exposure to each oil, the dual-oil formulation, or cisplatin. Four-parameter logistic models provided estimated IC_50_ values, and exact two-sided permutation tests were used for Spearman rank analyses. Results: The dual-oil formulation yielded estimated IC_50_ values of 1.42% *v/v* in B16F10 cells and 4.85% *v/v* in MRC-5 cells, corresponding to an apparent selectivity index of 3.41. The complete eight-concentration series was non-monotonic and showed no significant rank association (B16F10: ρs = −0.4286, exact *p* = 0.2992; MRC-5: ρs = −0.3810, exact *p* = 0.3599). In a post hoc analysis of the descending branch (≥0.37% *v*/*v*), the association was perfect in B16F10 cells (ρs = −1.0000, exact *p* = 0.0167) but did not reach significance in MRC-5 cells (ρs = −0.9000, exact *p* = 0.0833). The docking matrix identified target-dependent numerical prioritization patterns; however, small score differences were not interpreted as evidence of stronger binding or cellular target engagement. Conclusions: The findings define an exploratory chemical–computational–cellular framework and a preliminary formulation-level viability phenotype. They do not establish synergy, apoptosis, pathway modulation, therapeutic efficacy, or clinical safety. Independent multi-batch chemical confirmation, a fully documented and redocking-validated computational protocol, expanded melanoma and normal-skin cell panels, orthogonal functional assays, longer exposure periods, mechanistic validation, and subsequent in vivo studies are required.

## 1. Introduction

Cutaneous malignant melanoma is characterized by metastatic potential, marked molecular heterogeneity, phenotypic plasticity, and frequent resistance to targeted and immune-based treatment. The RAS–RAF–MEK–ERK axis and several parallel survival, stress-response, inflammatory, and lineage-associated programs contribute to disease progression and therapeutic adaptation [1,2,3,4,5,6,7,8,9,10,11,12,13,14,15,16,17,18,19,20,21,22,23,24]. These biological complexities also require careful separation of preliminary in vitro observations from claims of target engagement, antitumor mechanism, or clinical efficacy.

Natural products, essential oils, monoterpenes, polyphenols, and complex phytochemical mixtures have been investigated in melanoma and other tumor models because they can produce measurable effects on cellular viability, redox balance, mitochondrial function, apoptosis, proliferation, migration, and inflammatory signaling [8,9,13,14,15,16,17,18,19,20,21,22,23,24,25,26,27,28,29,30,31,32,33,34,35,36,37,38,39]. Nevertheless, natural origin does not imply selectivity, safety, reproducibility, or therapeutic value. Chemical composition, botanical provenance, extraction method, storage, vehicle behavior, concentration, exposure duration, assay interference, and the selected cellular model are critical determinants of interpretation [9,21,30,31,32,33,34,35,36,38,39].

α-Pinene and other monoterpenes found in Pinus-derived essential oils have shown biological activity in melanoma-relevant and other tumor systems [25,26,27,28,36,37,38,40,41,42,43,44,45,46,47,48]. *Prunus dulcis* oil is chemically distinct and is typically enriched in oleic, linoleic, and palmitic acids; Prunus-derived lipidic, phenolic, and terpenoid fractions have also been investigated in cancer models [49,50,51,52,53,54]. Recent analytical studies confirm that almond oils are generally oleic/linoleic-rich and that *Pinus sylvestris* needle essential oils commonly contain α-pinene, β-pinene, and carene-type monoterpenes, although substantial geographic and processing variability exists [52,53,54,55,56].

Within the literature surveyed for the present study, we did not identify a previous report that integrated study-sample GC/MS and FAME characterization of cold-pressed *Prunus dulcis* oil and *Pinus sylvestris* needle essential oil, comparator-context molecular docking of the principal identified markers, representative three-dimensional ligand–protein complexes, and side-by-side 24 h testing of each oil and their combination in B16F10 melanoma cells and MRC-5 fibroblasts. The principal novelty is therefore the integration of chemical characterization, computational prioritization, and comparative formulation-level viability assessment rather than demonstration of a molecular mechanism. This study aimed to characterize the two oil samples, contextualize structurally plausible ligand–target interactions, quantify the 24 h NRU viability phenotype of the individual and combined preparations, estimate IC_50_ and apparent selectivity indices, and evaluate concentration–viability ordering using exact rank-based statistics.

## 2. Materials and Methods

### 2.1. Study Design

This exploratory in vitro study combined chemical profiling of two oil samples with 24 h NRU viability testing in B16F10 melanoma cells and MRC-5 fibroblasts. Each oil was tested individually and in combination; cisplatin served as a reference cytotoxic comparator. This study was designed to characterize a formulation-level cellular phenotype and did not include mechanistic, pathway-specific, animal, or clinical endpoints.

### 2.2. Oil Samples, Formulation, and Provenance Limitations

The tested formulation contained cold-pressed *Prunus dulcis* oil and *Pinus sylvestris* needle essential oil. Single-oil preparations and the combined formulation were evaluated. Chemical identity and composition of the analyzed study samples were assessed by the chromatographic procedures described below. The available source records did not provide complete manufacturer/supplier, catalog, lot, geographic origin, production date, voucher specimen, certificate of analysis, storage history, oxidation index, or independently manufactured batch information. Accordingly, this study does not claim formal botanical authentication or batch-to-batch reproducibility.

### 2.3. GC/MS and GC/FID-Based FAME Analysis

Gas chromatography/mass spectrometry (GC/MS) analyses were performed using an Agilent Technologies (Stevens Creek Blvd, Santa Clara, CA, USA) 7890B gas chromatograph coupled to an Agilent Technologies (Stevens Creek Blvd, Santa Clara, CA, USA) inert selective 5977A mass selective detector from the same manufacturer. Separations were performed on a nonpolar HP-5MS silica capillary column (5% diphenyl- and 95% dimethyl-polysiloxane; 30 m × 0.25 mm i.d., 0.25 μm film thickness; Agilent Technologies, Santa Clara, CA, USA). Helium was used as the carrier gas at a constant flow rate of 1 mL/min. The MSD transfer line, ion source and quadrupole temperatures were set at 300 °C, 230 °C and 150 °C, respectively, and electron ionization was performed at 70 eV.

Prior to GC/MS and GC/FID analyses, fatty acids from almond oil were converted into the corresponding fatty acid methyl esters (FAMEs) by esterification/transesterification. The obtained FAMEs were dissolved in dichloromethane, and 1 μL of the prepared solution was injected through a split/splitless inlet maintained at 220 °C, operating in split mode at a split ratio of 100:1. The oven temperature was initially held at 150 °C for 5 min, increased to 250 °C at 10 °C/min and held for 10 min, then increased to 300 °C at 30 °C/min and finally held at 300 °C for 3 min. Mass spectra were recorded in scan mode over the range of *m*/*z* 10–600.

For essential oil analysis, the sample was dissolved in diethyl ether, and 1 μL of the prepared solution was injected at 220 °C in split mode at a split ratio of 40:1. The oven temperature was programmed from 60 °C to 246 °C at a rate of 3 °C/min. Mass spectra were recorded over the range of *m*/*z* 41–415.

Data processing was performed using MSD ChemStation Data Analysis software, revision F.01.00.1903, in combination with AMDIS, version 2.70, and NIST MS Search, version 2.0 g. FAMEs were identified by comparison of their mass spectra with those available in the Wiley, NIST and RTLPEST mass spectral libraries. Essential oil constituents were identified by comparison of their experimentally determined retention indices (RIexp), calculated using a homologous series of n-alkanes C8–C20, with literature values (RIlit) and by comparison of their mass spectra with those from Wiley 6, NIST2011 and RTLPEST3 libraries. For selected constituents, identification was additionally confirmed by co-injection with authentic standards.

GC/FID analyses were carried out under the same chromatographic conditions as the corresponding GC/MS analyses. The flow rates of the carrier gas (He), make-up gas (N_2_), fuel gas (H_2_) and oxidizing gas (air) were 1, 25, 30 and 400 mL/min, respectively, and the FID temperature was set at 300 °C.

Quantitative analysis of FAMEs was performed by GC/FID using calibration curves prepared from a certified reference mixture of fatty acid methyl esters (F.A.M.E. Mix RM-6, Sigma-Aldrich, St. Louis, MO, USA). A series of standard solutions was prepared in dichloromethane over the concentration range of 1.1–17.4 mg/mL. Calibration curves were constructed by plotting GC/FID peak areas against the concentrations of the FAME standard solutions. The quantitative composition of FAMEs was expressed as mg/mL.

Semi-quantitative analysis of FAMEs and essential oil constituents, expressed as relative content (%), was performed using the GC/FID peak area normalization method based on automatic integration of GC/FID signals without correction factors.

The available analytical record did not retain the exact esterification/transesterification reagent composition, reagent-to-sample ratio, reaction temperature and duration, number of independent sample preparations, complete blank/QC injection sequence, calibration equations, or prespecified library-match and retention-index acceptance thresholds. These missing operational details limit independent reproduction and are explicitly acknowledged rather than retrospectively reconstructed.

### 2.4. Molecular Docking and Three-Dimensional Comparator-Context Visualization

The archived computational dataset included six formulation-associated markers (α-pinene, β-pinene, δ-3-carene, oleic acid, linoleic acid, and palmitic acid) and eight targets (BRAF V600E, MEK1, ERK2, KIT, CDK4/6, PARP1, COX-2, and tyrosinase). The retained outputs comprised docking scores, within-target ranking, pose-level descriptors, reference-drug comparisons, and representative three-dimensional ligand–protein complexes. Target-specific reference scores were harmonized with the reference-drug matrix, and all ΔScore values were recalculated as ligand score minus the corresponding comparator score.

The available archive did not retain sufficient information to reconstruct the full docking protocol, including the complete receptor structure identifiers, receptor-preparation sequence, grid centers and dimensions, exhaustiveness, number of generated poses, ligand protonation and tautomeric states, treatment of crystallographic water molecules, software build and random seed, or co-crystallized-ligand redocking validation. No missing parameters were reconstructed retrospectively. The docking outputs are therefore reported as qualitative comparator-context evidence and not as a fully validated quantitative binding study. Numerical differences below approximately 1 kcal/mol were not interpreted as evidence of stronger binding.

### 2.5. Cell Lines and Neutral-Red Uptake Assay

Cytotoxicity was evaluated in MRC-5 fibroblasts and B16F10 melanoma cells using the neutral-red uptake assay. The *Prunus dulcis* and *Pinus sylvestris* oils were tested separately and in combination; cisplatin was used as a reference compound.

MRC-5 and B16F10 cells were seeded in 96-well plates at 1 × 10^4^ cells/well in 200 μL of high-glucose Dulbecco’s Modified Eagle Medium supplemented with 10% heat-inactivated fetal bovine serum, 2 mM glutamine, 100 U/mL penicillin, and 100 μg/mL streptomycin (Sigma-Aldrich, Darmstadt, Germany). Cells were incubated for 24 h at 37 °C in a humidified 5% CO_2_ atmosphere. The medium was then replaced with preparations containing the oils at 0.045%, 0.09%, 0.18%, 0.37%, 1%, 1.25%, 3%, or 5% *v*/*v* or cisplatin at 0.18, 0.5, 1, 2, 3, 4, 5, or 20 mM. Exposure continued for 24 h. Two independent experiments were performed, with triplicate technical measurements in each experiment.

One day before the assay, neutral-red working solution (Sigma-Aldrich, St. Louis, MO, USA) was prepared in DMEM at a 1:65 dilution, supplemented with 20 mM HEPES, and equilibrated overnight at 37 °C in 5% CO_2_. On the assay day, the solution was passed through a 0.2 μm sterile filter, and 150 μL was added per well.

After treatment, the medium was removed, and cells were washed twice with pre-warmed phosphate-buffered saline. Cells were incubated with neutral-red solution for 3 h at 37 °C in 5% CO_2_ and washed twice, and lysosomal dye was extracted with 150 μL of destaining solution (50% ethanol, 49% distilled water, and 1% glacial acetic acid; *v*/*v*/*v*) for 10 min at 300 rpm. Absorbance was measured at 540 nm with a 630 nm reference wavelength using an Agilent BioTek Epoch microplate spectrophotometer (Santa Clara, CA, USA). Viability was expressed relative to untreated controls.

### 2.6. Dose–Response Modeling and Statistical Analysis

Reported IC_50_ values were obtained by nonlinear four-parameter logistic modeling: Y = Bottom + (Top − Bottom)/[1 + 10^((logIC_50_ − X) × HillSlope)], where X is the logarithm of concentration. The apparent selectivity index was calculated as SI = IC_50_(MRC-5)/IC_50_(B16F10). Because complete replicate-level raw data and all model diagnostics were not available, IC_50_, SI, and reported between-cell-line *p* values were interpreted descriptively.

Spearman’s rank-order coefficient was calculated between concentration and mean NRU viability. Exact two-sided *p* values were obtained by enumerating all possible rank permutations (8! permutations for the complete series and 5! permutations for the post hoc descending branch). The complete eight-concentration analysis was primary. The ≥0.37% *v*/*v* branch was selected after visual inspection and was therefore labeled post hoc and exploratory. A nominal *p* < 0.05 threshold was used without treating the branch analysis as confirmatory.

## 3. Results

### 3.1. Study-Sample GC/MS and FAME Characterization

**Figure 1:**
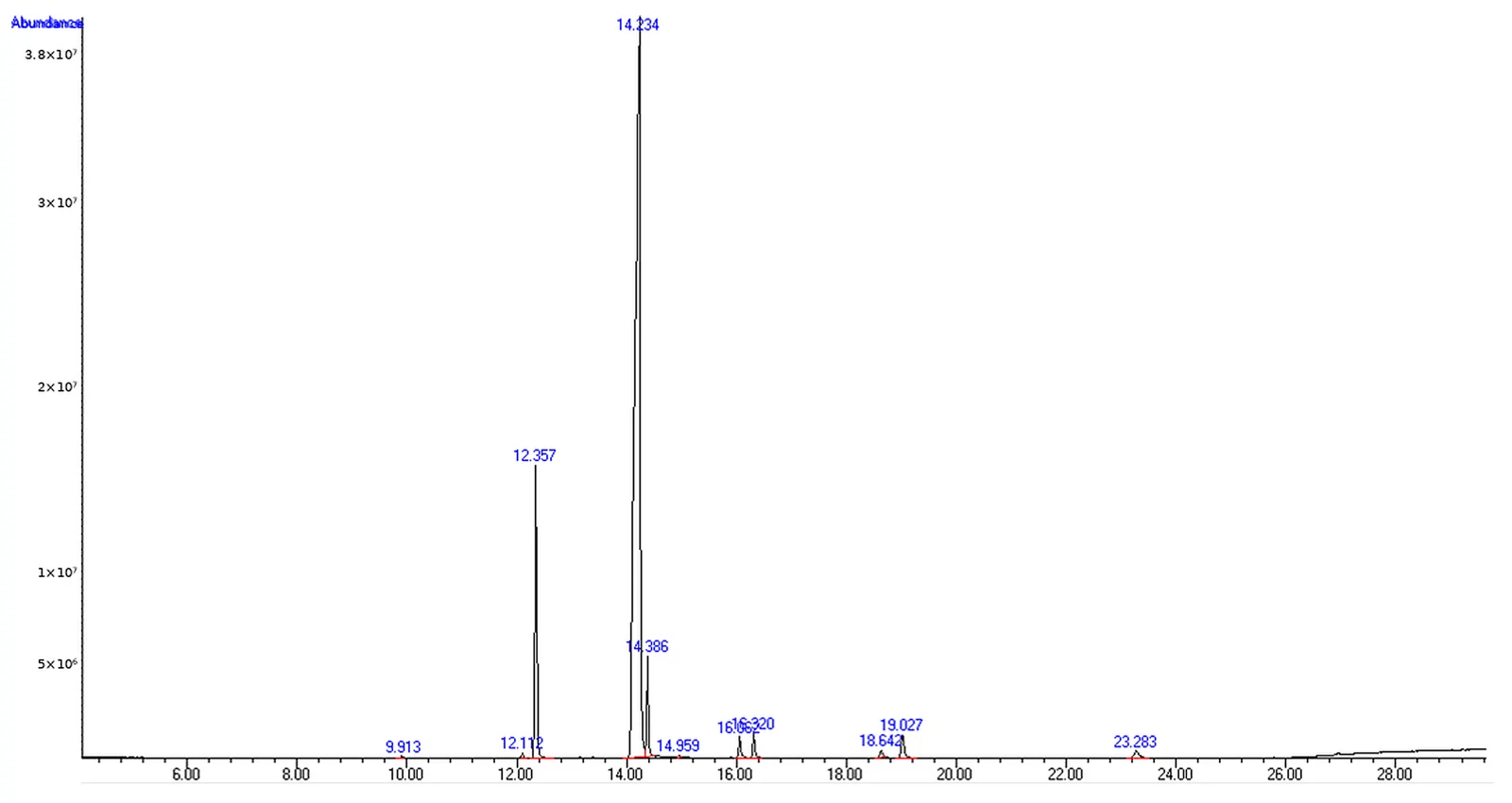
Representative GC/MS total-ion chromatogram of fatty acid methyl esters obtained from the analyzed cold-pressed *Prunus dulcis* oil sample. The black trace represents total-ion abundance, and blue annotations identify retention times (min). Closely spaced annotations correspond to adjacent chromatographic peaks; compound identities are reported in Table 2.

**Figure 2:**
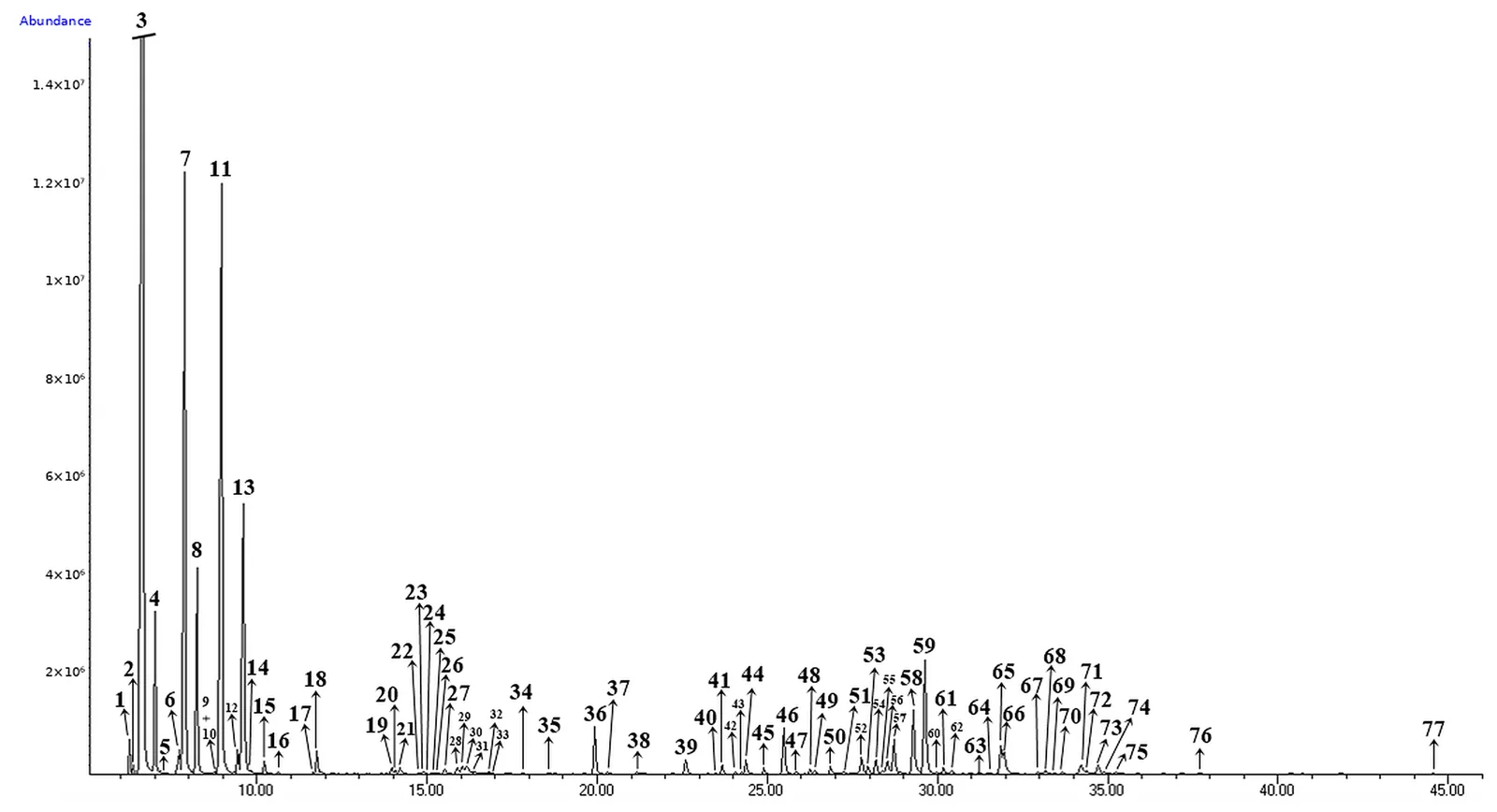
Representative GC/MS total-ion chromatogram of the analyzed *Pinus sylvestris* needle essential-oil sample. The black trace represents total-ion abundance. Peak numbers correspond directly to the compounds listed in Table 4; the blue axis labels denote instrument-generated chromatographic annotations.

The cold-pressed *Prunus dulcis* oil sample was dominated by oleic acid (52.7%), linoleic acid (27.9%), palmitic acid (11.0%), and stearic acid (3.2%) (Table 1 and Table 2; Figure 1). The *Pinus sylvestris* needle essential-oil sample contained 86.5% monoterpene hydrocarbons and was dominated by α-pinene (41.6%), δ-3-carene (14.8%), β-pinene (14.2%), limonene (7.0%), and myrcene (4.0%) (Table 3 and Table 4; Figure 2). These data establish the composition of the analyzed study samples; they do not, by themselves, demonstrate botanical authentication or batch-to-batch reproducibility.

The GC/MS and FAME profiles of the two analyzed oil samples were compared with recent analytical reports to establish compositional context and expected biological variability (Table 5).

### 3.2. Comparator-Context Molecular Docking Analysis

The docking dataset comprised six chemically identified formulation-associated markers—α-pinene, β-pinene, δ-3-carene, oleic acid, linoleic acid, and palmitic acid—evaluated across BRAF V600E, MEK1, ERK2, KIT, CDK4/6, PARP1, COX-2, and tyrosinase. The analysis was retained as a comparator-context layer linking the GC/MS/FAME profile to melanoma-relevant or melanoma-adjacent molecular nodes. Docking scores were used for within-target prioritization only and were not interpreted as biochemical inhibition, pharmacological potency, cellular target engagement, pathway modulation, or therapeutic efficacy. The complete target-by-ligand score matrix is summarized in Table 6.

Within each target, more negative scores were used to rank candidate ligands relative to the corresponding reference comparator. Because docking scoring functions have intrinsic uncertainty, numerical separations below approximately 1 kcal/mol were treated as indistinguishable for biological interpretation and were not described as evidence of superior binding. Table 7 therefore functions as a transparent prioritization matrix rather than as a potency ranking.

Pose-level descriptors were retained to document the internal structural annotations associated with the highest-ranked marker for each target. Hydrogen-bond counts, hydrophobic contacts, aromatic interactions, replicate-pose RMSD, interaction-fingerprint similarity, and pocket-position annotations are descriptive outputs and do not independently validate binding. These descriptors are summarized in Table 8.

The selected panel spans oncogenic signaling, cell-cycle control, DNA-damage response, inflammatory signaling, and melanocytic-lineage biology. This breadth is intended to provide a pathway-oriented hypothesis framework rather than to imply that the formulation modulates every target. The target ontology and inclusion rationale are summarized in Table 9.

Reference compounds were selected as recognizable pharmacological or mechanistic anchors for the corresponding target pockets. Their inclusion does not imply equivalence between the formulation-associated markers and clinically established inhibitors. The corresponding pharmacological anchors and binding-context assumptions are listed in Table 10.

**Figure 3:**
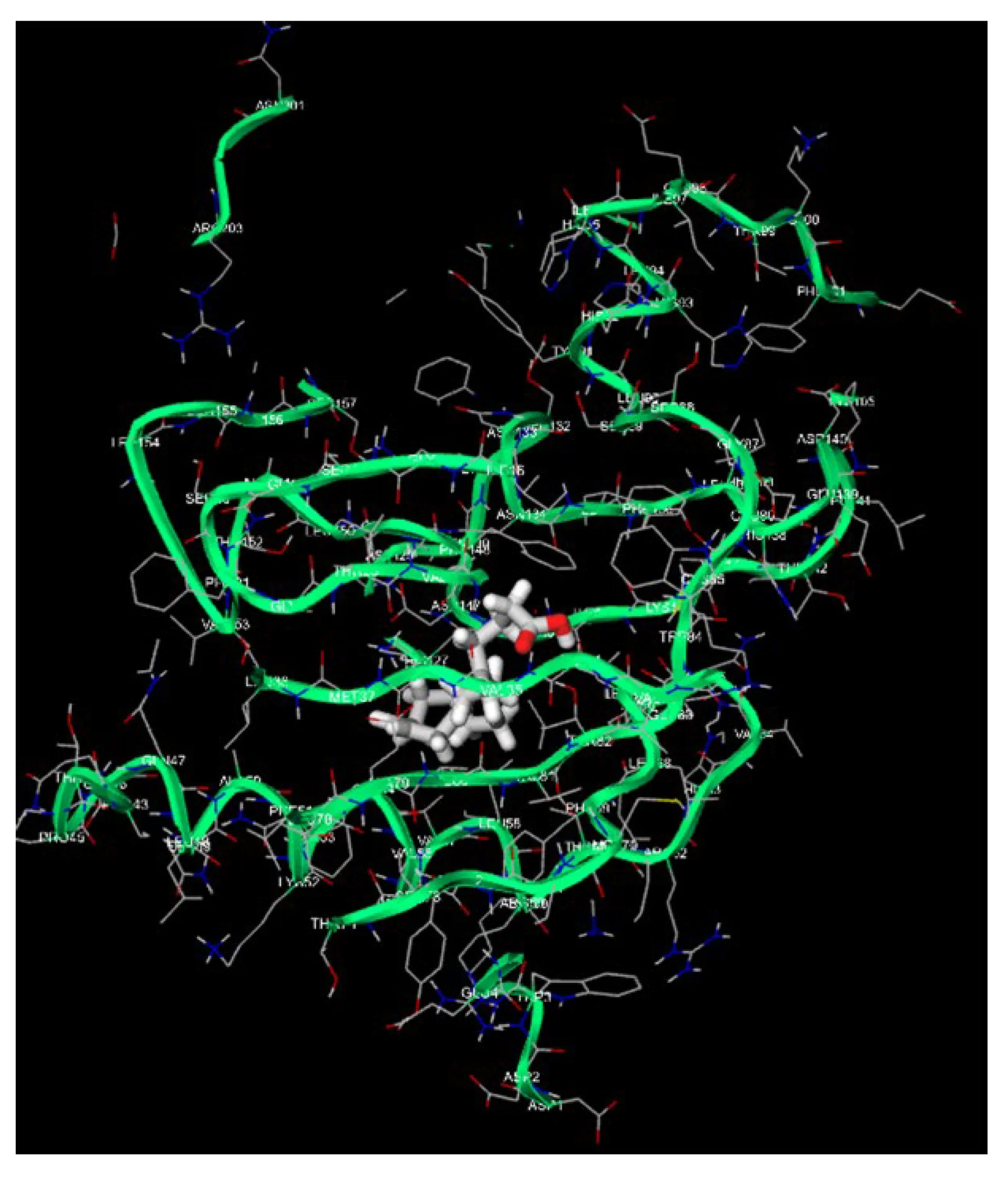
Three-dimensional docking pose and interaction environment of kojic acid in tyrosinase. The image contextualizes melanogenesis-related target geometry and does not demonstrate enzymatic inhibition by the formulation.

**Figure 4:**
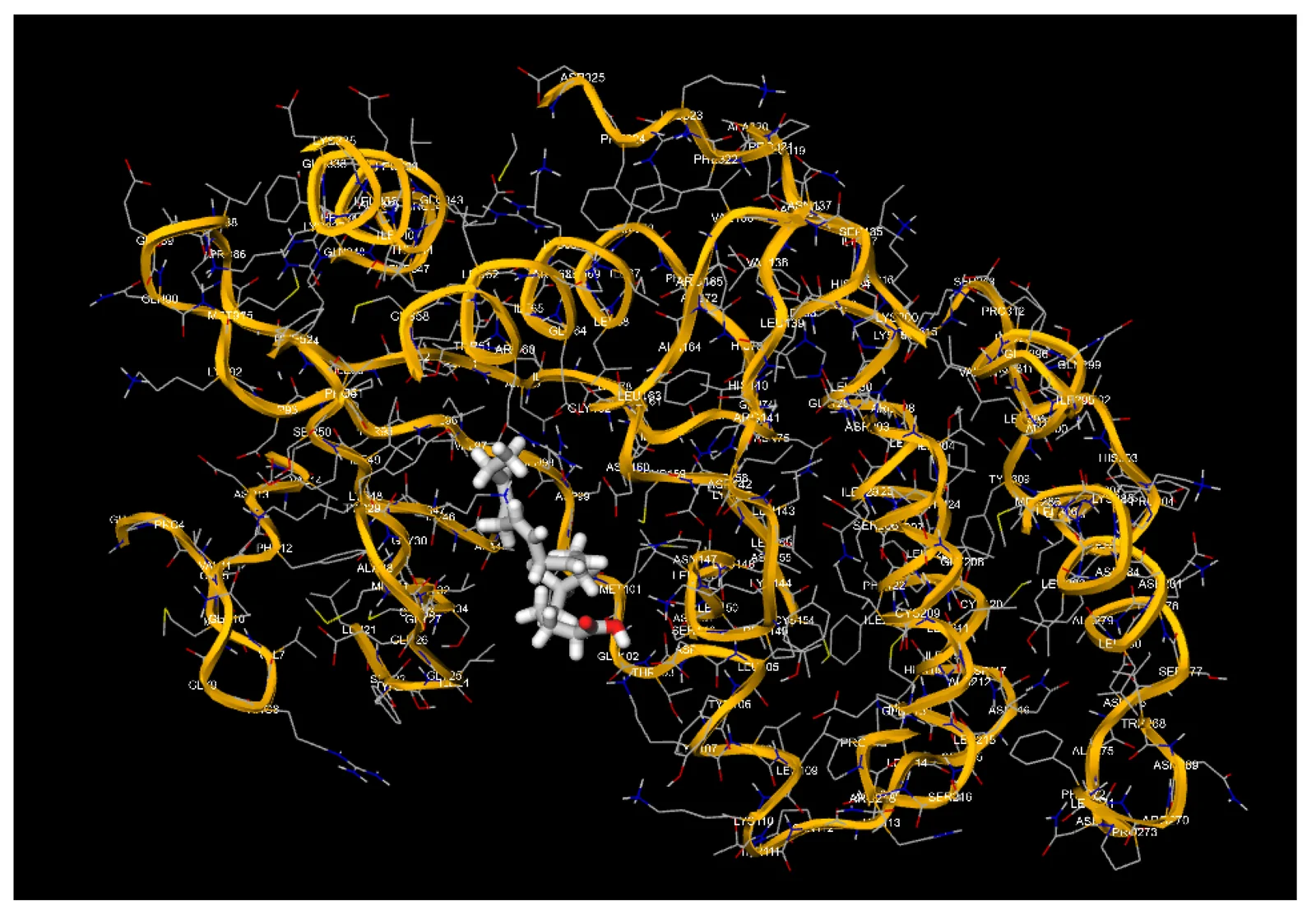
Representative three-dimensional comparator–ligand complex from the molecular-docking panel.

**Figure 5:**
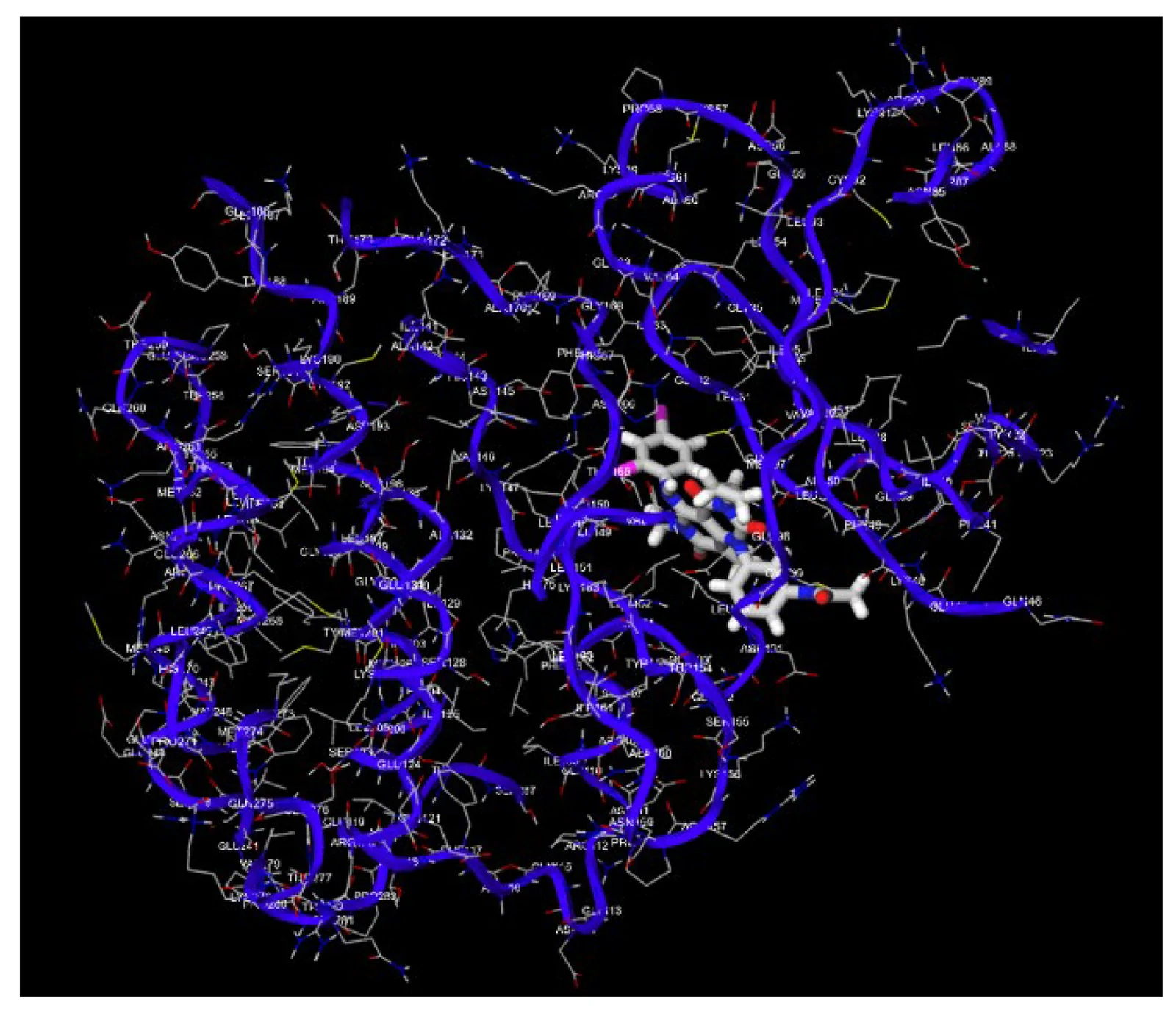
Representative three-dimensional complex of a prioritized formulation-associated ligand within a melanoma-relevant target pocket.

**Figure 6:**
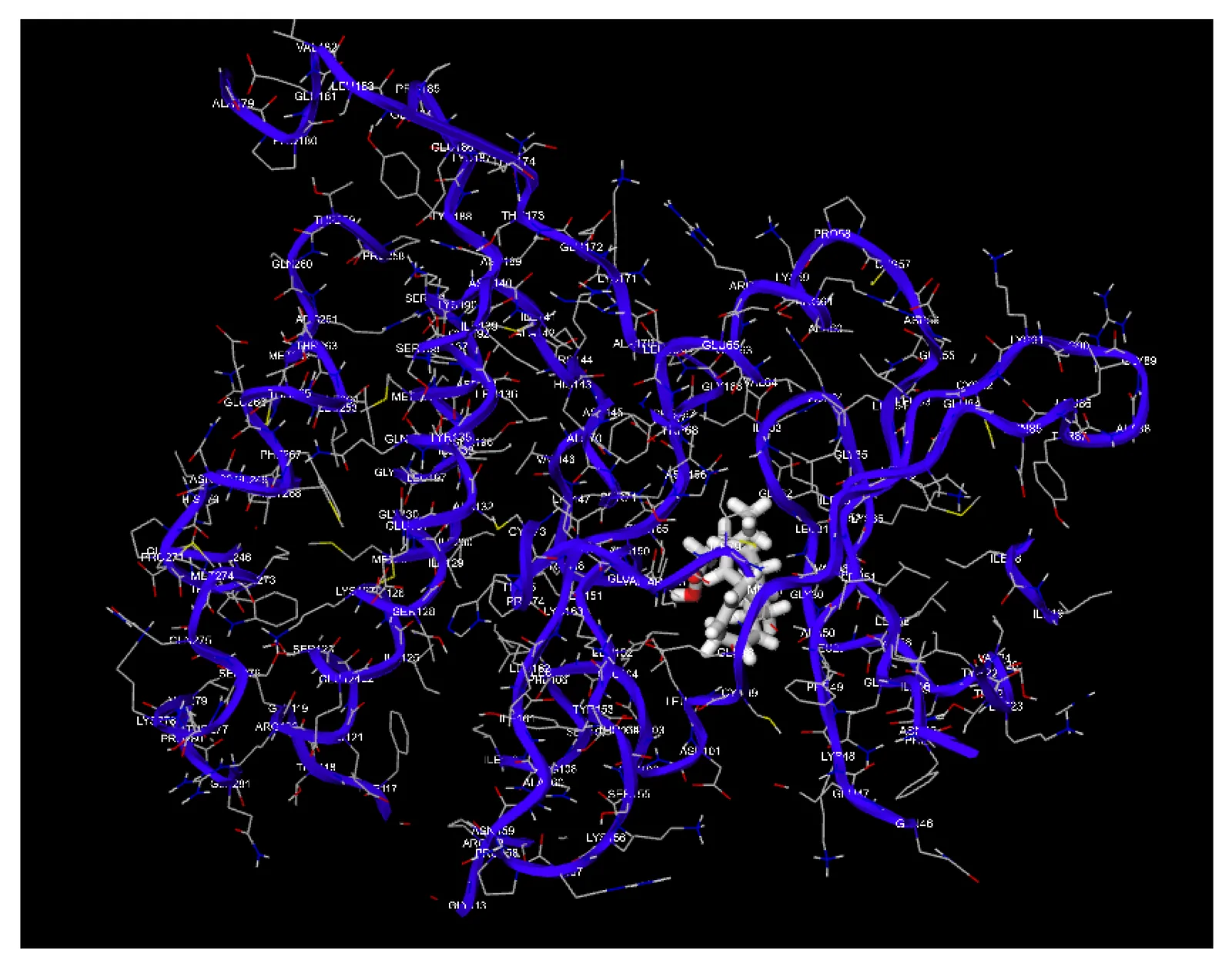
Representative three-dimensional comparator complex retained from the in silico target panel.

**Figure 7:**
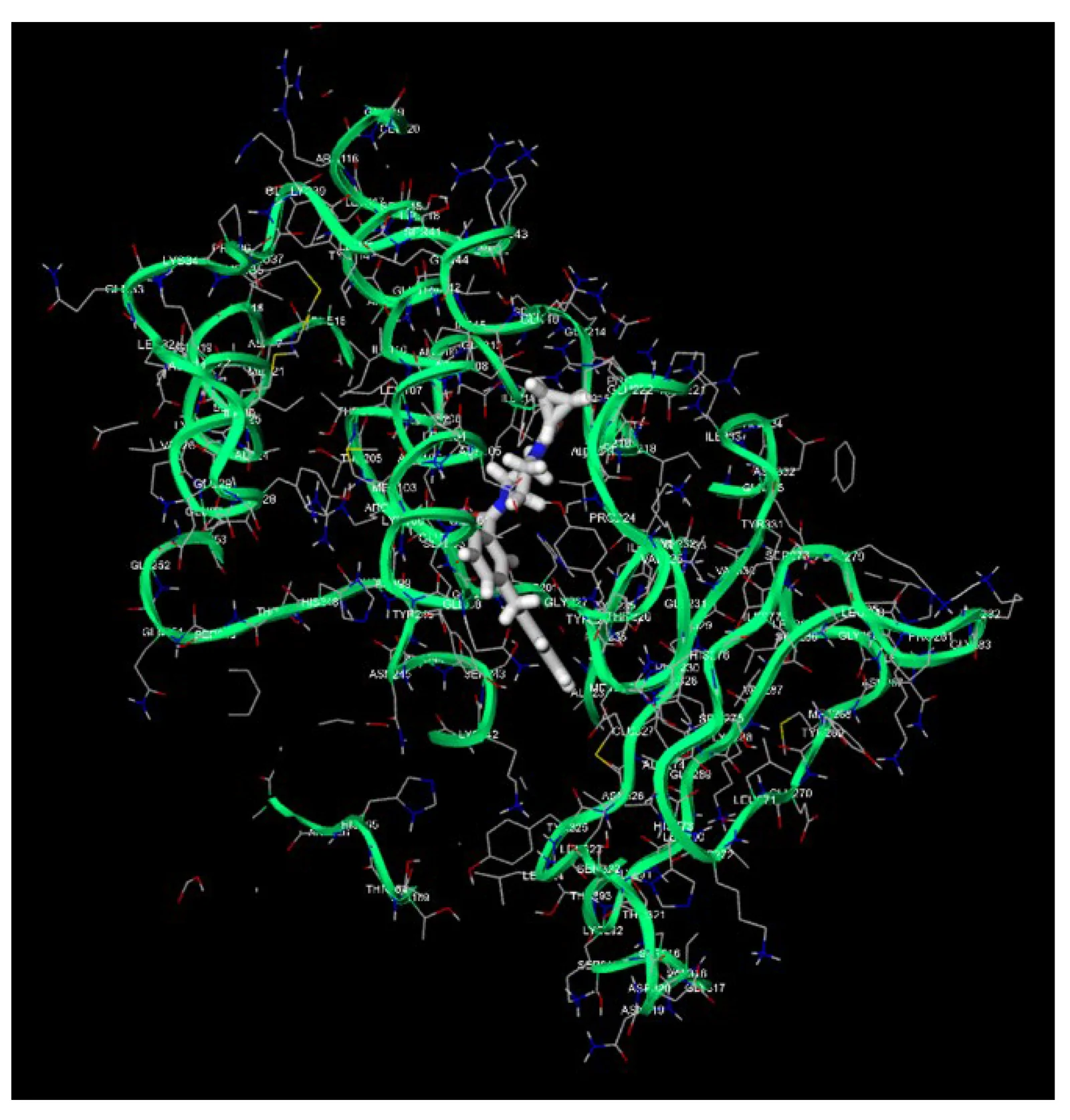
Additional three-dimensional docking visualization supporting qualitative structural-context interpretation.

**Figure 8:**
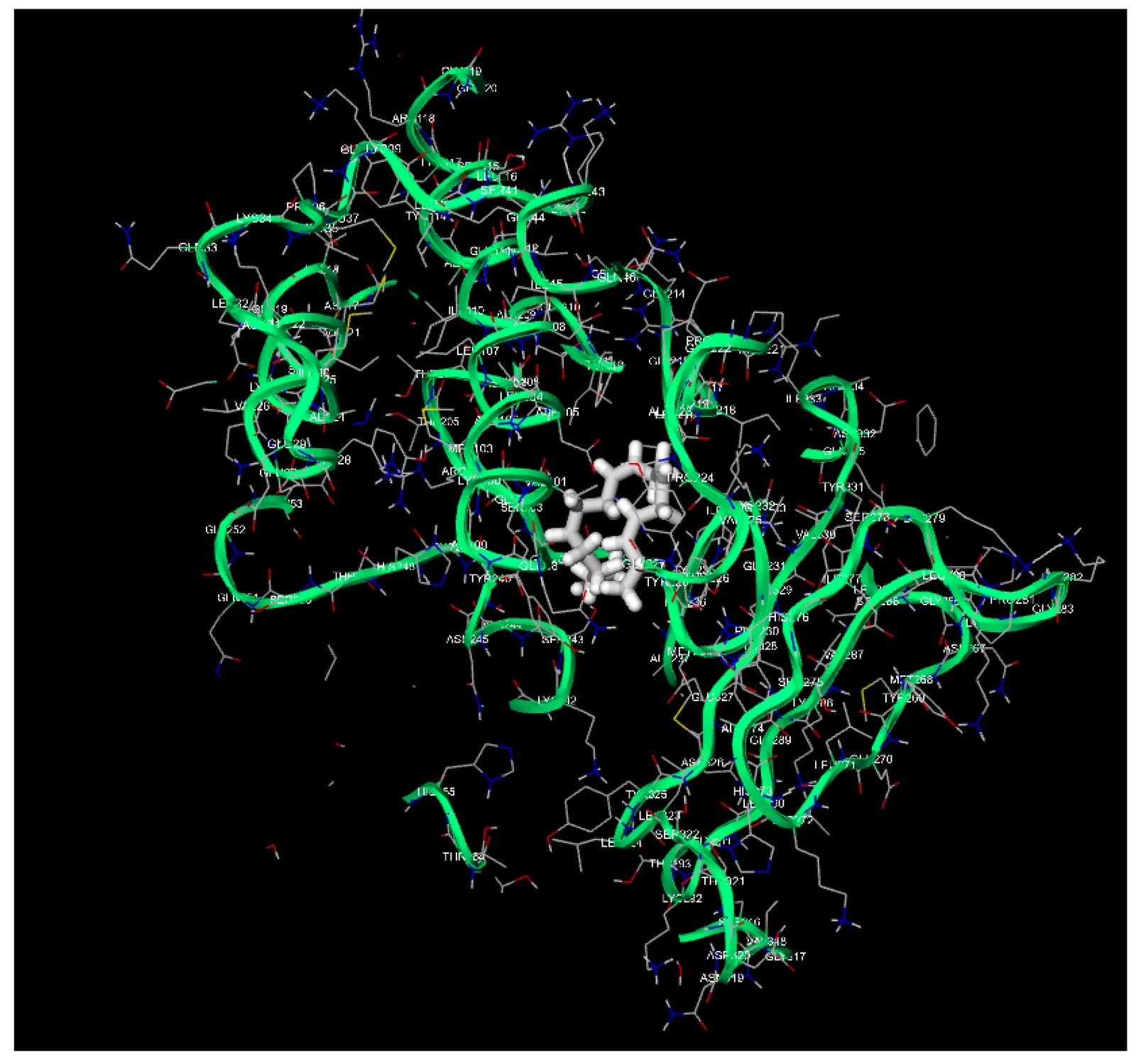
Additional three-dimensional docking visualization from the archived comparator-context analysis.

The α-pinene row was harmonized with Table 6 to remove the internal score discrepancy present in the earlier version. Table 11 places α-pinene, the predominant volatile constituent of the analyzed *Pinus sylvestris* sample, within the same target-by-target matrix as the reference compounds. Representative three-dimensional docking visualizations from the comparator-context analysis are presented in Figure 3, Figure 4, Figure 5, Figure 6, Figure 7 and Figure 8.

Representative three-dimensional complexes from the archived computational analysis are shown below. These images are presented as qualitative structural-context visualizations and are not evidence of target inhibition or intracellular engagement.

### 3.3. Twenty-Four-Hour NRU Viability Profiles

**Figure 9:**
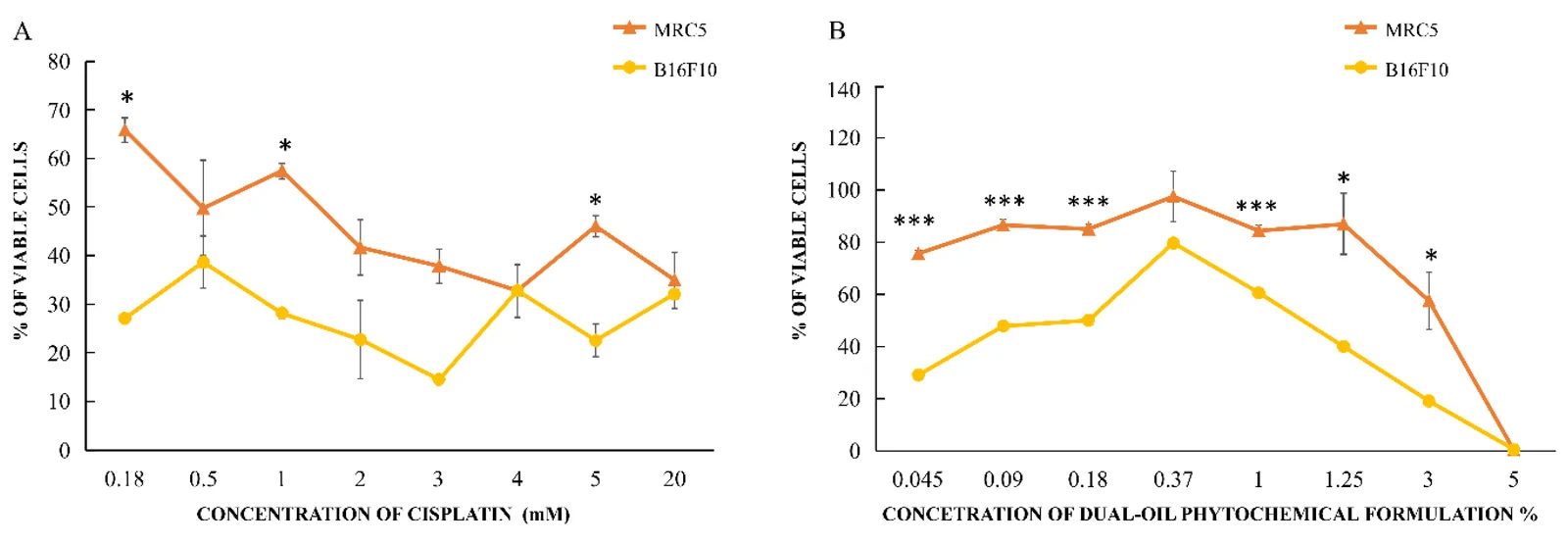
Twenty-four-hour NRU concentration–response profiles for (**A**) cisplatin and (**B**) the dual-oil formulation in B16F10 melanoma cells and MRC-5 fibroblasts. Values are presented as mean ± SE from two independent experiments with triplicate technical measurements. Reported between-cell-line comparisons are descriptive because complete replicate-level model diagnostics were not available. * *p* < 0.05; *** *p* < 0.001.

The combined formulation produced a non-monotonic concentration–viability profile in both cell lines (Figure 9; Table 7). Intermediate concentrations were associated with increased viability relative to adjacent concentrations, followed by a pronounced decline at higher exposures. At 3% *v/v*, mean viability was 18.96% in B16F10 cells and 57.41% in MRC-5 fibroblasts; at 5% *v/v*, viability approached zero in both cell lines. Accordingly, the fitted IC_50_ estimates should be interpreted as exploratory model-based summaries rather than as evidence of a uniformly monotonic dose response.

**Figure 10:**
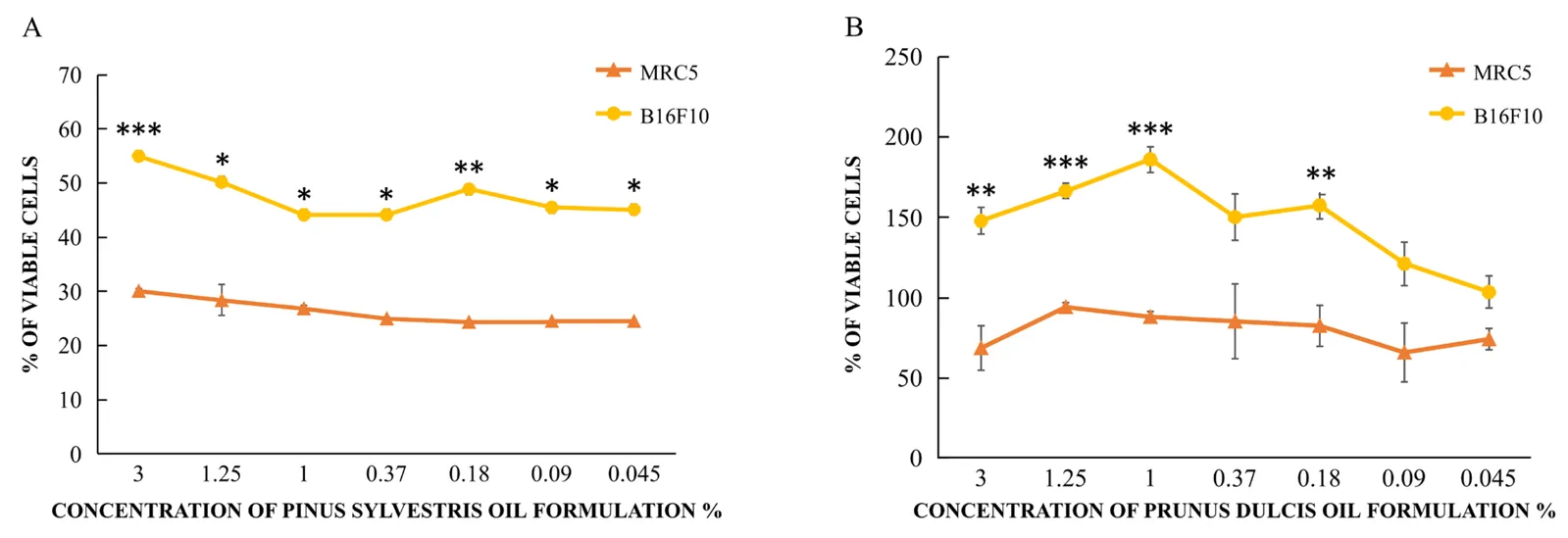
Twenty-four-hour NRU concentration–response profiles for the individual *Pinus sylvestris* (**A**) and *Prunus dulcis* (**B**) oil samples in B16F10 melanoma cells and MRC-5 fibroblasts. Values are presented as mean ± SE from two independent experiments with triplicate technical measurements. The statistic elaboration was based on these expressions of significance: * *p* < 0.05, ** *p* < 0.01, *** *p* < 0.001.

Testing of the individual oils showed that *Pinus sylvestris* essential oil reduced viability in both cell lines, with reported IC_50_ values of 1.34% *v/v* in B16F10 cells and 0.79% *v/v* in MRC-5 fibroblasts (SI = 0.59), indicating greater sensitivity of the fibroblast model and no tumor-selective pattern. *Prunus dulcis* oil did not reach an IC_50_ within the tested concentration range and was associated with increased NRU signal at several concentrations. Thus, neither single oil reproduced the apparent differential response estimated for the combined formulation (Figure 10). These data are insufficient to infer synergy because a factorial dose matrix and a formal interaction model were not used.

Cisplatin reduced viability in both B16F10 melanoma cells and MRC-5 fibroblasts, with B16F10 cells generally showing greater sensitivity at low-to-intermediate concentrations. The response was not strictly monotonic, and the difference between the two cell lines diminished at several higher concentrations. The reported descriptive *p* values indicate statistically distinguishable responses at selected concentrations, whereas other exposure levels showed comparable viability between the two cell lines (Table 12). The dual-oil formulation produced a markedly different viability profile between B16F10 melanoma cells and MRC-5 fibroblasts across most tested concentrations. B16F10 viability was generally lower than MRC-5 viability, indicating greater melanoma-cell sensitivity, although the concentration–response pattern was non-monotonic. At the highest concentration (5% *v/v*), viability was almost completely suppressed in both cell lines. The reported descriptive *p* values further indicate concentration-dependent differences between the two cellular models (Table 13). The reported 4PL estimates were 1.42% *v*/*v* for B16F10 cells and 4.85% *v*/*v* for MRC-5 fibroblasts, yielding an apparent SI of 3.41 (Table 14). This value indicates differential sensitivity in the two tested cell lines under the specified 24 h NRU conditions, but it does not establish general tumor selectivity because only one non-malignant fibroblast line was examined. Cisplatin yielded reported IC_50_ values of 0.74 mM and 1.21 mM in B16F10 and MRC-5 cells, respectively (SI = 1.63); direct potency comparisons between cisplatin and the oils remain limited by different units, mechanisms, and concentration ranges.

### 3.4. Exact Spearman Rank Analysis

**Figure 11:**
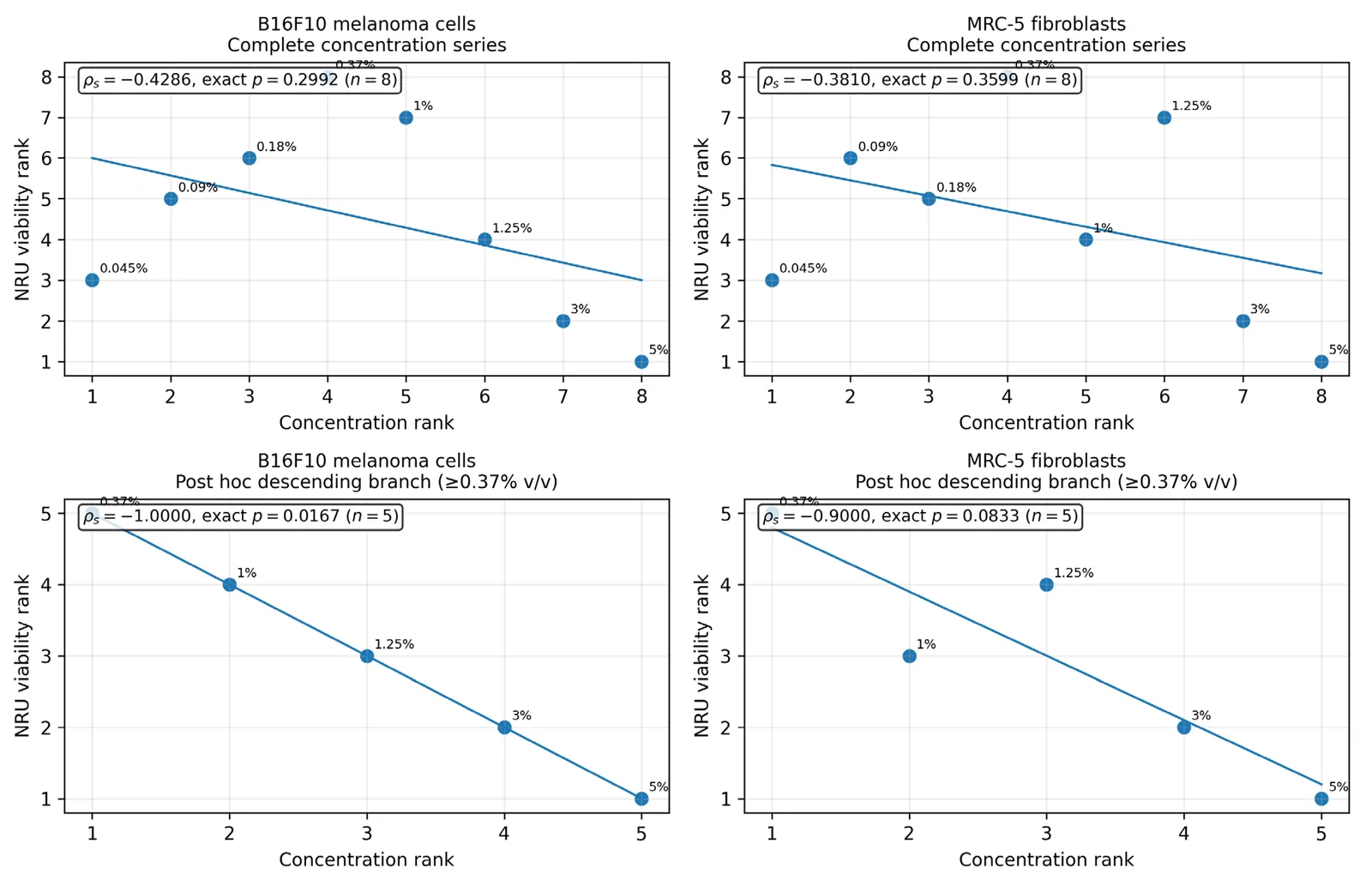
Exact Spearman rank analysis of dual-oil concentration and 24 h NRU viability. Upper panels show the complete eight-point series; lower panels show the post hoc descending branch (≥0.37% *v/v*; *n* = 5). The blue line is a least-squares visual trend across ranked observations and is included only as a visual guide; inferential interpretation is based on Spearman ρs and exact two-sided permutation *p* values obtained by enumerating all rank permutations.

Across all eight dual-oil concentrations, concentration and mean NRU viability were inversely but non-significantly ranked in B16F10 cells (ρs = −0.4286, exact two-sided *p* = 0.2992) and MRC-5 fibroblasts (ρs = −0.3810, exact two-sided *p* = 0.3599). Because visual inspection showed a descending response from 0.37% to 5% *v/v*, a post hoc sensitivity analysis was performed for this five-point branch. The branch showed ρs = −1.0000 with exact *p* = 0.0167 in B16F10 cells and ρs = −0.9000 with exact *p* = 0.0833 in MRC-5 cells (Figure 11). The B16F10 result met the nominal 0.05 threshold, whereas the MRC-5 result did not. Both branch analyses remain exploratory because the interval was selected after inspection and was not an independently replicated primary endpoint.

## 4. Discussion

### 4.1. Principal Findings and Study Novelty

This study integrates chemical characterization of the analyzed oils, comparator-context molecular docking of the principal identified markers, representative three-dimensional ligand–protein visualizations, and side-by-side 24 h NRU testing of the individual and combined preparations. The dual-oil formulation produced a lower estimated IC_50_ in B16F10 melanoma cells than in MRC-5 fibroblasts, whereas *Pinus sylvestris* oil alone was more toxic to fibroblasts and *Prunus dulcis* oil did not reach an IC_50_. Within the literature surveyed, this chemical–computational–cellular integration constitutes the main novelty. The novelty is methodological and hypothesis-generating; it does not demonstrate a new antitumor mechanism.

### 4.2. Chemical Context and Comparison with Previous Reports

The fatty-acid profile of the *Prunus dulcis* sample was consistent with recent analyses of almond cultivars and genotypes, which report oleic and linoleic acids as dominant components, with palmitic and stearic acids at lower abundance [49,50,51,52,53,54]. The *Pinus sylvestris* sample was similarly consistent with monoterpene-rich Scots pine and related Pinus essential oils, in which α-pinene, β-pinene, 3-carene/carenes, limonene, and other monoterpenes frequently predominate [36,38,45,46,47,48,55,56]. This concordance supports the chemical plausibility of the analyzed samples but does not replace formal botanical authentication, oxidation testing, or independent batch replication.

### 4.3. Biological Interpretation in Relation to Melanoma Literature

α-Pinene and other monoterpenes have been associated with apoptosis, mitochondrial perturbation, oxidative stress, and reduced tumor growth in melanoma-relevant or other cancer models [40,41,42,43,44]. Pinus-derived fractions and essential oils have also shown cytotoxic or pro-apoptotic effects in B16F10 and other tumor systems [36,37,38,45,46,47,48]. In parallel, *Prunus dulcis* oil and other Prunus-derived fractions have shown composition-dependent biological activity in non-melanoma cancer models [49,50,51]. Essential-oil phytocomplexes such as frankincense, oregano, and *Melaleuca alternifolia* preparations have been examined in melanoma models using apoptosis, oxidative stress, DNA damage, and in vivo endpoints [25,26,27,28,29]. These studies provide biological context but cannot be used to assign the same mechanisms to the present formulation.

The current experiment measured lysosomal neutral-red accumulation after 24 h. Reduced NRU signal may reflect loss of viable cell number, lysosomal dysfunction, metabolic stress, membrane effects, or assay–matrix interactions; it does not by itself distinguish apoptosis, necrosis, cytostasis, altered proliferation, or impaired clonogenic survival. Likewise, no measurements of MAPK, PARP1, COX-2, CDK4/6, KIT, tyrosinase, inflammatory cytokines, oxidative stress, migration, or invasion were performed. Any pathway-level interpretation must therefore remain a hypothesis for future testing [13,23,37,57,58,59,60,61,62,63,64].

### 4.4. Computational Interpretation and Target-Validation Boundaries

The docking results provide a structured means of prioritizing ligand–target pairs for subsequent biochemical testing. Target-dependent ranking was observed, with α-pinene, β-pinene, δ-3-carene, linoleic acid, oleic acid, and palmitic acid occupying different numerical positions across BRAF V600E, MEK1, ERK2, KIT, CDK4/6, PARP1, COX-2, and tyrosinase. These differences are compatible with variation in pocket geometry and ligand physicochemical properties, but they cannot establish affinity, selectivity, inhibition, or pathway modulation. In particular, score separations below approximately 1 kcal/mol were treated as being within the practical uncertainty of the scoring function [62,63,64].

The archived docking outputs did not preserve the complete information required for independent reproduction and formal validation, including all receptor identifiers, grid centers and dimensions, exhaustiveness, generated-pose counts, protonation and tautomeric states, crystallographic-water handling, random seeds, and co-crystallized-ligand redocking results. Consequently, the computational layer is presented as descriptive structural context. Experimental assessment by enzyme assays, Western blotting, qPCR, phosphorylation analysis, or orthogonal target-engagement methods is required before any pathway-level inference can be made.

### 4.5. Selectivity, Interaction Claims, and Principal Limitations

The apparent SI of 3.41 is based on one murine melanoma line and one human fetal-lung fibroblast line. It should not be generalized to normal skin because primary melanocytes, keratinocytes, dermal fibroblasts, immune cells, and reconstructed skin models were not tested. The MRC-5 comparison provides an initial non-malignant reference only. Furthermore, formal synergy cannot be inferred from the present single-concentration-series design. Bliss, Loewe, Highest Single Agent, ZIP, or Chou–Talalay analysis would require a factorial matrix of independently varied component concentrations.

Additional limitations include the use of a single viability assay, a single 24 h time point, two independent experiments, incomplete replicate-level model diagnostics, non-monotonic concentration–response behavior, no human melanoma line, no clonogenic or migration/invasion endpoint, no pathway validation, and no in vivo efficacy or safety assessment. Essential oils may also cause irritation, oxidative stress, or inflammatory responses that are not captured by fibroblast viability alone. The available source records did not fully document producer, lot, geographic origin, voucher authentication, production date, oxidation indices, or the number of independently manufactured oil batches. Consequently, the reported chemistry applies to the analyzed study samples and does not establish batch-to-batch consistency.

### 4.6. Future Validation Priorities

Future work should first establish provenance and chemical reproducibility across independently sourced batches, including replicated GC/MS/FAME profiling, oxidation and stability indices, and prespecified analytical quality-control criteria. Cellular studies should then test the individual major constituents and reconstituted mixtures in murine and human melanoma lines together with primary melanocytes, keratinocytes, and dermal fibroblasts. Orthogonal endpoints should include ATP- or protein-based viability assays, live/dead imaging, Annexin V/propidium iodide analysis, caspase activation, mitochondrial membrane potential, reactive oxygen species, cell-cycle distribution, clonogenic survival, and migration/invasion assays at 24, 48, and 72 h.

Only after reproducible functional effects are demonstrated should mechanistic studies evaluate candidate pathways by Western blotting, qPCR, enzyme assays, or phosphoprotein profiling. Any future computational prioritization should use a fully documented and validated protocol, including receptor identifiers, ligand and receptor preparation, grid definitions, search parameters, co-crystallized-ligand redocking, and pose-reproduction criteria [62,63,64]. Controlled skin-compatibility and inflammatory-response testing should precede animal studies. In vivo melanoma efficacy, pharmacokinetics, local tolerability, and systemic safety would be required before any clinical application could be considered.

## 5. Conclusions

The analyzed *Prunus dulcis* and *Pinus sylvestris* oil samples showed the expected fatty-acid-rich and monoterpene-rich chemical profiles, respectively. The comparator-context docking dataset provided target-dependent numerical prioritization and representative three-dimensional structural models, while the combined formulation produced an exploratory 24 h NRU phenotype with a lower estimated IC_50_ in B16F10 melanoma cells than in MRC-5 fibroblasts and an apparent SI of 3.41. The complete concentration series was non-monotonic and not significant by exact Spearman testing. The post hoc descending branch was significant in B16F10 cells but not in MRC-5 fibroblasts. These findings support further controlled investigation but do not establish stronger molecular binding, cellular target engagement, synergy, apoptosis, pathway modulation, broad tumor selectivity, therapeutic efficacy, or clinical safety.

Future studies should prioritize independent multi-batch chemical validation, complete provenance and stability documentation, a fully specified molecular-docking workflow with co-crystallized-ligand redocking and predefined pose-reproduction criteria, purified-marker and reconstituted-mixture testing, expanded human melanoma and normal-skin cell panels, orthogonal functional assays, 48–72 h and clonogenic endpoints, formal interaction modeling, mechanistic pathway measurements, and skin-compatibility testing. In vivo melanoma efficacy and safety studies should be considered only after chemical, computational, and cellular reproducibility have been established.

## Figures and Tables

**Table 1:** Quantitative and relative composition of the principal fatty acids in the analyzed *Prunus dulcis* oil sample, determined as fatty acid methyl esters.

Fatty Acid	FAME	Content (mg/mL)	Relative Content (%)
Oleic acid	Methyl oleate	30.66	52.7
Linoleic acid	Methyl linoleate	14.54	27.9
Palmitic acid	Methyl palmitate	6.12	11.0
Stearic acid	Methyl stearate	1.75	3.2
Palmitoleic acid	Methyl palmitoleate	0.12	0.2
Myristic acid	Methyl myristate	0.04	0.1

**Table 2:** Complete fatty-acid profile of the analyzed *Prunus dulcis* oil sample determined by GC/MS analysis of fatty acid methyl esters.

No.	tR (min)	Fatty Acid (Identified as Methyl Ester)	RIexp	RIlit	Identification Method	Content (%)
1.	9.93	Myristic acid (as methyl myristate)	1729	1726 ^a^	RI, MS, Co-I	tr
2.	12.07	(7E)-Hexadecenoic acid (as methyl ester)	1905	1900 ^h^	RI, MS	tr
3.	12.13	Palmitoleic acid (as methyl palmitoleate)	1910	1912 ^b^	RI, MS, Co-I	0.2
4.	12.36	Palmitic acid (as methyl palmitate)	1931	1928 ^c^	RI, MS, Co-I	11.0
5.	13.17	(Z)-Heptadec-10-enoic acid (as methyl ester)	2007	2016 ^d^	RI, MS	tr
6.	13.40	Margaric acid (as methyl margarate)	2031	2030 ^f^	RI, MS	tr
7.	14.20	Linoleic acid (as methyl linoleate)	2107	2104 ^d^	RI, MS, Co-I	27.9
8.	14.25	Oleic acid (as methyl oleate)	2117	2109 ^d^	RI, MS, Co-I	52.7
9.	14.41	Stearic acid (as methyl stearate)	2134	2135 ^e^	RI, MS, Co-I	3.2
10.	16.08	11-Eicosenoic acid (as methyl ester)	2309	2310 ^d^	RI, MS	1.0
11.	16.34	Arachidic acid (as methyl arachidate)	2331	2332 ^f^	RI, MS	1.0
12.	18.64	(Z)-13-Docosenoic acid (erucic acid, as methyl ester)	2509	2508 ^d^	RI, MS	0.5
13.	19.07	Behenic acid (as methyl behenate)	2532	2530 ^g^	RI, MS	1.6
14.	23.37	Lignoceric acid (as methyl lignocerate)	2733	2731 ^g^	RI, MS	0.9
Total identified (%) = 100.0						

*Notes: RIexp, experimentally determined retention index; RIlit, literature retention index; RI, retention-index match; MS, mass-spectral match; Co-I, co-injection with an authentic standard; tr, trace. Superscript letters a–h identify literature-source codes retained in the archived analytical export; the corresponding source-code key was not preserved and was therefore not reconstructed retrospectively*.

**Table 3:** Major constituents and grouped chemical composition of the analyzed *Pinus sylvestris* needle essential-oil sample determined by GC/MS.

Compound	Content (%)	Compound	Content (%)
α-Pinene	41.6	δ-Cadinene	2.5
δ-3-Carene	14.8	Camphene	2.4
β-Pinene	14.2	γ-Cadinene	1.5
Limonene	7.0	(E)-Caryophyllene	1.1
Myrcene	4.0	Other identified constituents	10.9
Grouped components (%)			Content (%)
Monoterpene hydrocarbons			86.5
Sesquiterpene hydrocarbons			8.6
Oxygen-containing monoterpenes			2.5
Oxygen-containing sesquiterpenes			2.2
Diterpenes			0.1
Others			0.1
Total identified (%)			100.0

**Table 4:** Complete chemical composition of the analyzed *Pinus sylvestris* needle essential-oil sample determined by GC/MS.

No.	tR (min)	Compound	RIexp	RIlit	Method of Identification	Content (%)
1.	6.26	Tricyclene	918	921 ^a^	RI, MS	0.5
2.	6.37	α-Thujene	921	924 ^a^	RI, MS	0.1
3.	6.66	α-Pinene	932	932 ^a^	RI, MS	41.6
4.	7.01	Camphene	944	946 ^a^	RI, MS	2.4
5.	7.15	Thuja-2,4(10)-diene	949	953 ^a^	RI, MS	0.1
6.	7.72	Sabinene	968	969 ^a^	RI, MS	0.6
7.	7.89	β-Pinene	974	974 ^a^	RI, MS, Co-I	14.2
8.	8.24	Myrcene	986	988 ^a^	RI, MS	4.0
9.	8.74	α-Phellandrene	1003	1002 ^a^	RI, MS	0.1
10.	8.78	(3E)-Hexenyl acetate	1004	1001 ^a^	RI, MS	tr
11.	8.96	δ-3-Carene	1009	1008 ^a^	RI, MS	14.8
12.	9.44	o-Cymene	1021	1022 ^a^	RI, MS	0.4
13.	9.59	Limonene	1026	1024 ^a^	RI, MS, Co-I	7.0
14.	9.62	β-Phellandrene	1026	1025 ^a^	RI, MS	tr
15.	10.21	(E)-β-Ocimene	1042	1044 ^a^	RI, MS	0.3
16.	10.62	γ-Terpinene	1054	1054 ^a^	RI, MS, Co-I	tr
17.	11.65	p-Mentha-2,4(8)-diene	1081	1085 ^a^	RI, MS	tr
18.	11.76	Terpinolene	1084	1086 ^a^	RI, MS	0.4
19.	13.96	trans-Pinocarveol	1138	1135 ^a^	RI, MS	0.2
20.	14.07	Camphor	1141	1441 ^a^	RI, MS, Co-I	0.1
21.	14.20	trans-Verbenol	1144	1143 ^b^	RI, MS	0.2
22.	14.73	trans-Pinocamphone	1157	1158 ^a^	RI, MS	tr
23.	14.81	Pinocarvone	1159	1160 ^a^	RI, MS	tr
24.	14.89	p-Mentha-1,5-dien-8-ol	1161	1166 ^a^	RI, MS	0.1
25.	15.12	Borneol	1166	1165 ^a^	RI, MS	tr
26.	15.30	cis-Pinocamphone	1170	1172 ^a^	RI, MS	tr
27.	15.54	Terpinen-4-ol	1176	1174 ^a^	RI, MS, Co-I	0.1
28.	15.87	m-Cymen-8-ol	1184	1176 ^a^	RI, MS	0.2
29.	16.05	p-Cymen-8-ol	1188	1179 ^a^	RI, MS	0.2
30.	16.16	α-Terpineol	1191	1186 ^a^	RI, MS	0.2
31.	16.39	Myrtenol	1197	1194 ^a^	RI, MS	tr
32.	16.72	p-Cymen-9-ol	1204	1204 ^a^	RI, MS	tr
33.	16.82	Verbenone	1207	1204 ^a^	RI, MS	tr
34.	17.81	Carvacrol, methyl ether	1231	1241 ^a^	RI, MS	tr
35.	18.58	Car-3-en-2-one	1248	1244 ^a^	RI, MS	tr
36.	19.94	Isobornyl acetate	1280	1283 ^a^	RI, MS	0.9
37.	20.31	2-Undecanone	1288	1288 ^c^	RI, MS	0.1
38.	21.16	Carvacrol	1308	1298 ^a^	RI, MS	tr
39.	22.59	α-Terpinyl acetate	1343	1346 ^a^	RI, MS	0.4
40.	23.46	α-Ylangene	1363	1373 ^a^	RI, MS	tr
41.	23.66	α-Copaene	1369	1374 ^a^	RI, MS	0.2
42.	24.05	β-Bourbonene	1378	1387 ^a^	RI, MS	0.1
43.	24.25	β-Cubebene	1382	1387 ^a^	RI, MS	0.1
44.	24.36	β-Elemene	1385	1389 ^a^	RI, MS	0.3
45.	24.89	Longifolene	1398	1407 ^a^	RI, MS	0.1
46.	25.47	(E)-Caryophyllene	1412	1417 ^a^	RI, MS, Co-I	1.1
47.	25.86	β-Copaene	1422	1430 ^a^	RI, MS	0.1
48.	26.24	Aromadendrene	1432	1439 ^a^	RI, MS	0.1
49.	26.39	6,9-Guaiadiene	1435	1442 ^a^	RI, MS	0.1
50.	26.85	α-Humulene	1447	1452 ^a^	RI, MS	0.2
51.	27.25	cis-Muurola-4(14),5-diene	1456	1465 ^a^	RI, MS	0.1
52.	27.76	γ-Muurolene	1469	1478 ^a^	RI, MS, Co-I	0.4
53.	27.95	Germacrene D	1474	1480 ^a^	RI, MS	0.2
54.	28.17	β-Selinene	1479	1489 ^a^	RI, MS	0.3
55.	28.39	trans-Muurola-4(14),5-diene	1485	1493 ^a^	RI, MS	0.1
56.	28.51	α-Selinene	1488	1498 ^a^	RI, MS	0.3
57.	28.71	α-Muurolene	1493	1500 ^a^	RI, MS	0.7
58.	29.28	γ-Cadinene	1507	1513 ^a^	RI, MS	1.5
59.	29.62	δ-Cadinene	1516	1522 ^a^	RI, MS	2.5
60.	29.98	trans-Cadina-1,4-diene	1525	1533 ^a^	RI, MS	tr
61.	30.17	α-Cadinene	1530	1537 ^a^	RI, MS	0.1
62.	30.42	α-Calacorene	1537	1544 ^a^	RI, MS	0.1
63.	31.19	β-Calacorene	1557	1564 ^a^	RI, MS	tr
64.	31.49	1α,10α-epoxy-Amorph-4-ene	1564	1570 ^a^	RI, MS	tr
65.	31.85	Spathulenol	1574	1577 ^a^	RI, MS	0.7
66.	31.94	Caryophyllene oxide	1577	1582 ^a^	RI, MS	0.7
67.	32.96	Humulene epoxide II	1603	1608 ^a^	RI, MS	0.1
68.	33.16	1,10-di-epi-Cubenol	1609	1618 ^a^	RI, MS	0.1
69.	33.43	α-Corocalene	1616	1622 ^a^	RI, MS	tr
70.	33.67	1-epi-Cubenol	1622	1627 ^a^	RI, MS	tr
71.	34.21	epi-α-Cadinol	1637	1638 ^a^	RI, MS	0.3
72.	34.36	α-Muurolol	1641	1644 ^a^	RI, MS	0.1
73.	34.70	α-Cadinol	1651	1652 ^a^	RI, MS	0.2
74.	34.90	cis-Calamenen-10-ol	1656	1660 ^a^	RI, MS	tr
75.	35.22	trans-Calamenen-10-ol	1664	1668 ^a^	RI, MS	tr
76.	37.69	Oplopanone	1733	1739 ^a^	RI, MS	tr
77.	44.55	Cembrene	1937	1937 ^a^	RI, MS	0.1
Total identified (%) = 100.0						
Grouped components (%)						Content (%)
Monoterpene hydrocarbons (1–9, 11–18)						86.5
Oxygen-containing monoterpenes (19–36, 38, 39)						2.5
Sesquiterpene hydrocarbons (40–63, 69)						8.6
Oxygen-containing sesquiterpenes (64–68, 70–76)						2.2
Diterpenes (77)						0.1
Others (10, 37)						0.1

Superscript letters identify literature-source codes retained in the archived analytical export.

**Table 5:** Recent literature comparators used to contextualize the GC/MS and FAME profiles of the analyzed *Prunus dulcis* and *Pinus sylvestris* oil samples.

References	Matrix/Species	Analytical Approach	Main Comparable Compositional Data	Relevance to Present GC-MS/FAME Profile
[52]	Almond cultivars (*Prunus amygdalus* L.)	GC-FID fatty-acid profiling	Oleic acid up to 57.24%; linoleic acid 26.15%; palmitic acid 11.63%; stearic acid 3.65%	Highly concordant with the present almond oil profile: oleic acid 52.7%, linoleic acid 27.9%, palmitic acid 11.0%, stearic acid 3.2%.
[53]	*Prunus dulcis* genotypes from Yozgat, Türkiye	Fatty-acid profiling	Oleic acid 45.47–64.06%; linoleic acid 23.32–38.90%; palmitic acid 6.11–7.92%	Supports the expected dominance of oleic and linoleic acids and confirms that the present almond oil falls within a plausible compositional window.
[54]	Almond oil extracted/recovered by environmentally friendly methods	LC-MS lipidomics and fatty-acid profiling	18:1 72–75%; 18:2 22–25%; 16:0 4–5%	Provides a processing-focused comparator showing a more oleic-rich profile, useful for discussing cultivar/process variability.
[55]	*Pinus sylvestris* needles from Central Kazakhstan	GC-MS essential-oil analysis	Major constituents included α-pinene, β-pinene, camphene, τ-cadinol, and caryophyllene; minor constituents included limonene, 3-carene, and caryophyllene oxide	Directly supports the presence of α-pinene, β-pinene, limonene, 3-carene/carenes and caryophyllene-related constituents in Scots pine needle essential oils.
[56]	Pinus essential oils from selected species, including *Pinus sylvestris*	Review of chemical composition and bioactivity	Across Pinus species, α-pinene is consistently dominant; needles frequently contain limonene, β-pinene, δ-3-carene, bornyl acetate, (E)-caryophyllene and myrcene	Provides broader Pinus essential-oil context for the monoterpene-rich profile observed in the present *Pinus sylvestris* oil.

**Table 6:** GC-MS/FAME-guided docking scores (kcal/mol) of representative formulation-associated markers across the comparator-context target panel. Target-specific reference values were harmonized with the archived target-specific reference-drug matrix.

Ligand	BRAF V600E	MEK1	ERK2	KIT	CDK4/6	PARP1	COX-2	Tyrosinase
α-Pinene (Pinus sylvestris EO; GC-MS 41.6%)	−10.12	−8.76	−8.18	−7.99	−7.50	−6.06	−9.14	−7.44
β-Pinene (Pinus sylvestris EO; GC-MS 14.2%)	−9.27	−7.86	−8.46	−7.97	−9.30	−7.74	−8.99	−9.61
δ-3-Carene (Pinus sylvestris EO; GC-MS 14.8%)	−7.80	−6.89	−7.74	−8.80	−7.91	−8.16	−7.27	−8.91
Oleic acid (Prunus dulcis oil; FAME 52.7%)	−7.61	−7.85	−8.12	−8.09	−8.58	−8.53	−7.67	−7.92
Linoleic acid (Prunus dulcis oil; FAME 27.9%)	−8.44	−9.84	−8.71	−8.45	−9.00	−9.03	−8.12	−8.27
Palmitic acid (Prunus dulcis oil; FAME 11.0%)	−9.29	−9.03	−8.31	−7.32	−9.15	−6.99	−8.44	−9.30
Within-target reference comparator	−6.10	−7.80	−8.10	−6.70	−7.70	−8.20	−5.90	−8.10

**Table 7:** Corrected within-target ligand ranking and ΔScore values relative to the harmonized target-specific reference comparator. ΔScore was calculated as ligand score minus comparator score; negative values indicate a numerically more favorable docking score, without establishing stronger binding.

Target	Ligand	Docking Score (kcal/mol)	Within-Target Comparator	ΔScore vs. Comparator (kcal/mol)
BRAF V600E	α-Pinene (*Pinus sylvestris* EO; GC-MS 41.6%)	−10.12	Vemurafenib (−6.10)	−4.02
BRAF V600E	Palmitic acid (*Prunus dulcis* oil; FAME 11.0%)	−9.29	Vemurafenib (−6.10)	−3.19
BRAF V600E	β-Pinene (*Pinus sylvestris* EO; GC-MS 14.2%)	−9.27	Vemurafenib (−6.10)	−3.17
BRAF V600E	Linoleic acid (*Prunus dulcis* oil; FAME 27.9%)	−8.44	Vemurafenib (−6.10)	−2.34
BRAF V600E	δ-3-Carene (*Pinus sylvestris* EO; GC-MS 14.8%)	−7.80	Vemurafenib (−6.10)	−1.70
BRAF V600E	Oleic acid (*Prunus dulcis* oil; FAME 52.7%)	−7.61	Vemurafenib (−6.10)	−1.51
MEK1	Linoleic acid (*Prunus dulcis* oil; FAME 27.9%)	−9.84	Trametinib (−7.80)	−2.04
MEK1	Palmitic acid (*Prunus dulcis* oil; FAME 11.0%)	−9.03	Trametinib (−7.80)	−1.23
MEK1	α-Pinene (*Pinus sylvestris* EO; GC-MS 41.6%)	−8.76	Trametinib (−7.80)	−0.96
MEK1	β-Pinene (*Pinus sylvestris* EO; GC-MS 14.2%)	−7.86	Trametinib (−7.80)	−0.06
MEK1	Oleic acid (*Prunus dulcis* oil; FAME 52.7%)	−7.85	Trametinib (−7.80)	−0.05
MEK1	δ-3-Carene (*Pinus sylvestris* EO; GC-MS 14.8%)	−6.89	Trametinib (−7.80)	0.91
ERK2	Linoleic acid (*Prunus dulcis* oil; FAME 27.9%)	−8.71	Ulixertinib (−8.10)	−0.61
ERK2	β-Pinene (*Pinus sylvestris* EO; GC-MS 14.2%)	−8.46	Ulixertinib (−8.10)	−0.36
ERK2	Palmitic acid (*Prunus dulcis* oil; FAME 11.0%)	−8.31	Ulixertinib (−8.10)	−0.21
ERK2	α-Pinene (*Pinus sylvestris* EO; GC-MS 41.6%)	−8.18	Ulixertinib (−8.10)	−0.08
ERK2	Oleic acid (*Prunus dulcis* oil; FAME 52.7%)	−8.12	Ulixertinib (−8.10)	−0.02
ERK2	δ-3-Carene (*Pinus sylvestris* EO; GC-MS 14.8%)	−7.74	Ulixertinib (−8.10)	0.36
KIT	δ-3-Carene (*Pinus sylvestris* EO; GC-MS 14.8%)	−8.80	Imatinib (−6.70)	−2.10
KIT	Linoleic acid (*Prunus dulcis* oil; FAME 27.9%)	−8.45	Imatinib (−6.70)	−1.75
KIT	Oleic acid (*Prunus dulcis* oil; FAME 52.7%)	−8.09	Imatinib (−6.70)	−1.39
KIT	α-Pinene (*Pinus sylvestris* EO; GC-MS 41.6%)	−7.99	Imatinib (−6.70)	−1.29
KIT	β-Pinene (*Pinus sylvestris* EO; GC-MS 14.2%)	−7.97	Imatinib (−6.70)	−1.27
KIT	Palmitic acid (*Prunus dulcis* oil; FAME 11.0%)	−7.32	Imatinib (−6.70)	−0.62
CDK4/6	β-Pinene (*Pinus sylvestris* EO; GC-MS 14.2%)	−9.30	Palbociclib (−7.70)	−1.60
CDK4/6	Palmitic acid (*Prunus dulcis* oil; FAME 11.0%)	−9.15	Palbociclib (−7.70)	−1.45
CDK4/6	Linoleic acid (*Prunus dulcis* oil; FAME 27.9%)	−9.00	Palbociclib (−7.70)	−1.30
CDK4/6	Oleic acid (*Prunus dulcis* oil; FAME 52.7%)	−8.58	Palbociclib (−7.70)	−0.88
CDK4/6	δ-3-Carene (*Pinus sylvestris* EO; GC-MS 14.8%)	−7.91	Palbociclib (−7.70)	−0.21
CDK4/6	α-Pinene (*Pinus sylvestris* EO; GC-MS 41.6%)	−7.50	Palbociclib (−7.70)	0.20
PARP1	Linoleic acid (*Prunus dulcis* oil; FAME 27.9%)	−9.03	Olaparib (−8.20)	−0.83
PARP1	Oleic acid (*Prunus dulcis* oil; FAME 52.7%)	−8.53	Olaparib (−8.20)	−0.33
PARP1	δ-3-Carene (*Pinus sylvestris* EO; GC-MS 14.8%)	−8.16	Olaparib (−8.20)	0.04
PARP1	β-Pinene (*Pinus sylvestris* EO; GC-MS 14.2%)	−7.74	Olaparib (−8.20)	0.46
PARP1	Palmitic acid (*Prunus dulcis* oil; FAME 11.0%)	−6.99	Olaparib (−8.20)	1.21
PARP1	α-Pinene (*Pinus sylvestris* EO; GC-MS 41.6%)	−6.06	Olaparib (−8.20)	2.14
COX-2	α-Pinene (*Pinus sylvestris* EO; GC-MS 41.6%)	−9.14	Celecoxib (−5.90)	−3.24
COX-2	β-Pinene (*Pinus sylvestris* EO; GC-MS 14.2%)	−8.99	Celecoxib (−5.90)	−3.09
COX-2	Palmitic acid (*Prunus dulcis* oil; FAME 11.0%)	−8.44	Celecoxib (−5.90)	−2.54
COX-2	Linoleic acid (*Prunus dulcis* oil; FAME 27.9%)	−8.12	Celecoxib (−5.90)	−2.22
COX-2	Oleic acid (*Prunus dulcis* oil; FAME 52.7%)	−7.67	Celecoxib (−5.90)	−1.77
COX-2	δ-3-Carene (*Pinus sylvestris* EO; GC-MS 14.8%)	−7.27	Celecoxib (−5.90)	−1.37
Tyrosinase	β-Pinene (*Pinus sylvestris* EO; GC-MS 14.2%)	−9.61	Kojic acid (−8.10)	−1.51
Tyrosinase	Palmitic acid (*Prunus dulcis* oil; FAME 11.0%)	−9.30	Kojic acid (−8.10)	−1.20
Tyrosinase	δ-3-Carene (*Pinus sylvestris* EO; GC-MS 14.8%)	−8.91	Kojic acid (−8.10)	−0.81
Tyrosinase	Linoleic acid (*Prunus dulcis* oil; FAME 27.9%)	−8.27	Kojic acid (−8.10)	−0.17
Tyrosinase	Oleic acid (*Prunus dulcis* oil; FAME 52.7%)	−7.92	Kojic acid (−8.10)	0.18
Tyrosinase	α-Pinene (*Pinus sylvestris* EO; GC-MS 41.6%)	−7.44	Kojic acid (−8.10)	0.66

**Table 8:** Pose-level internal descriptors for top-ranked GC-MS/FAME-aligned phytochemical markers.

Target	Top Natural Ligand	H-Bonds (Count)	Hydrophobic Contacts (Count)	π–π/π–Cation (Count)	Pose RMSD vs. Replicates (Å)	IFP Similarity vs. Reference (0–1)	Occupancy of Back-Pocket/Channel
BRAF^V600E	α-Pinene (Pinus sylvestris EO; GC-MS 41.6%)	2	16	0	1.11	0.7	Hinge-adjacent
MEK1	Linoleic acid (Prunus dulcis oil; FAME 27.9%)	2	21	1	1.87	0.73	Solvent-front
ERK2	Linoleic acid (Prunus dulcis oil; FAME 27.9%)	2	18	0	0.79	0.72	Solvent-front
KIT	δ-3-Carene (Pinus sylvestris EO; GC-MS 14.8%)	1	19	2	1.28	0.6	Solvent-front
CDK4/6	β-Pinene (Pinus sylvestris EO; GC-MS 14.2%)	2	17	2	0.73	0.68	Channel
PARP1	Linoleic acid (Prunus dulcis oil; FAME 27.9%)	1	21	2	1.88	0.61	Back pocket
COX-2	α-Pinene (Pinus sylvestris EO; GC-MS 41.6%)	0	14	2	1.41	0.73	Back pocket
Tyrosinase	β-Pinene (Pinus sylvestris EO; GC-MS 14.2%)	0	19	1	0.57	0.41	Hinge-adjacent

**Table 9:** Melanoma-relevant target ontology and rationale for comparator-context inclusion.

Marker/Target	Pathway/Axis	Mechanistic Role in MM	Rationale for Inclusion
BRAF^V600E	MAPK (RAF→MEK→ERK)	Constitutive ERK drive; proliferative signaling	Primary oncogenic driver; SOC inhibitor benchmark
MEK1	MAPK	Signal relay to ERK; resistance node post-BRAF blockade	Allosteric druggable pocket; combination anchor
ERK2	MAPK	Terminal effector; transcriptional rewiring	Escape route upon upstream inhibition
KIT	RTK rebound	Upstream reactivation of MAPK/PI3K	Resistance adaptation; hinge-adjacent lipophilic shelves
CDK4/6	Cell-cycle	G1/S transition enforcement	Proliferative licensing; combination target
PARP1	DNA repair	DNA-damage tolerance via PARylation	Stress adaptation node; trench-like cavity
COX-2	Inflammation/prostanoids	Pro-inflammatory tone; microenvironmental support	Arachidonate channel compatibility with LCUFAs
Tyrosinase	Melanogenesis	Melanin biosynthesis; melanosomal biology	Potential substrate competition; gorge occupancy

**Table 10:** Reference inhibitors and binding-context assumptions used in the docking framework.

Target	Reference Drug	Mechanism/Class	Binding Topology	Contextual Note
BRAF^V600E	Vemurafenib (primary comparator); dabrafenib considered as an additional BRAF benchmark	ATP-competitive RAF inhibitor	Hinge binder + back-pocket occupancy	Primary BRAF comparator in the main docking set; dabrafenib used only as a contextual secondary benchmark
MEK1	Trametinib (primary comparator); cobimetinib considered as an additional MEK benchmark	Allosteric MEK inhibitor	Allosteric pocket vestibule	Primary MEK1 comparator in the main docking set; cobimetinib retained only for contextual interpretation
ERK2	Ulixertinib	ATP-competitive ERK inhibitor	Hinge + solvent-front	Terminal MAPK effector
KIT	Imatinib	ATP-competitive RTK inhibitor	Hinge-adjacent hydrophobic wall	RTK rebound mitigation
CDK4/6	Palbociclib/Ribociclib	ATP-competitive CDK inhibitor	Selective kinase hinge + back cleft	Proliferative licensing control
PARP1	Olaparib	NAD^+^-mimetic PARP inhibitor	Nicotinamide trench interactions	DNA-repair rheostat
COX-2	Celecoxib	COX-2 selective inhibitor	Arachidonate channel occupancy	Inflammatory tone modulation
Tyrosinase	Kojic acid	Active-site modulator	Chelation/aromatic stacking region	Melanogenesis attenuation

**Table 11:** Harmonized α-pinene and reference-drug docking scores across the comparator-context target panel.

Ligand/Drug	BRAF V600E	CDK4/6	COX-2 (PTGS2)	ERK2 (MAPK1)	KIT (CD117)	MEK1 (MAP2K1)	PARP1	Tyrosinase (TYR)
α-Pinene (*Pinus sylvestris*; GC-MS 41.6%)	−10.12	−7.50	−9.14	−8.18	−7.99	−8.76	−6.06	−7.44
Vemurafenib	−6.1	−5.7	−6.6	−5.4	−7.1	−6.1	−6.3	−6.9
Dabrafenib	−8.1	−7.8	−7.7	−7.5	−7.1	−8.2	−7.2	−8.5
Trametinib	−7.2	−7.1	−6.1	−7.6	−6.4	−7.8	−7.8	−6.6
Cobimetinib	−6.4	−7.1	−7.1	−7.8	−8.0	−6.9	−6.6	−5.9
Ulixertinib (investigational)	−7.9	−7.8	−8.2	−8.1	−8.6	−7.9	−7.8	−8.5
Imatinib	−6.6	−7.1	−6.9	−6.1	−6.7	−5.9	−7.1	−6.4
Palbociclib	−7.8	−7.7	−7.8	−7.7	−7.7	−7.9	−7.7	−8.0
Ribociclib	−6.9	−6.9	−7.7	−6.8	−6.8	−7.4	−7.5	−7.6
Olaparib	−7.9	−8.0	−8.5	−8.1	−8.0	−8.4	−8.2	−8.1
Celecoxib	−6.1	−6.2	−5.9	−6.5	−6.1	−5.7	−6.3	−6.4
Kojic acid (reference inhibitor)	−8.0	−8.0	−8.4	−8.4	−8.2	−8.1	−8.0	−8.1

**Table 12:** NRU viability after 24 h exposure to cisplatin. Data are presented as mean ± SE; reported *p* values are descriptive.

Cell Line/Statistic	0.18	0.5	1	2	3	4	5	20
MRC-5	65.86 ± 2.48	49.79 ± 9.78	57.39 ± 1.60	41.76 ± 5.69	37.82 ± 3.50	32.78 ± 0.94	46.00 ± 2.19	34.90 ± 5.84
B16F10	27.09 ± 0.51	38.62 ± 5.40	28.18 ± 1.09	22.67 ± 8.06	14.56 ± 0.69	32.74 ± 5.43	22.48 ± 3.35	32.05 ± 2.92
Reported *p* value	*p* = 0.034	*p* = 0.447	*p* = 0.007	*p* = 0.207	*p* = 0.085	*p* = 0.996	*p* = 0.039	*p* = 0.718

**Table 13:** NRU viability after 24 h exposure to the dual-oil formulation. Data are presented as mean ± SE; reported *p* values are descriptive.

Cell Line/Statistic	0.045	0.09	0.18	0.37	1	1.25	3	5
MRC-5	75.68 ± 1.10	86.59 ± 2.18	85.09 ± 1.60	97.45 ± 9.76	84.38 ± 2.09	86.96 ± 11.86	57.41 ± 10.98	0.25 ± 0.03
B16F10	28.99 ± 0.87	47.78 ± 1.20	50.00 ± 1.88	79.50 ± 4.05	60.51 ± 1.51	39.96 ±5.33	18.96 ± 6.40	0.34 ± 0.02
Reported *p* value	*p* < 0.001	*p* < 0.001	*p* < 0.001	*p* = 0.112	*p* < 0.001	*p* = 0.043	*p* = 0.011	*p* = 0.057

**Table 14:** Reported four-parameter logistic IC_50_ estimates and apparent selectivity indices.

Treatment	B16F10 IC_50_	MRC-5 IC_50_	Apparent SI
Dual-oil formulation: *Prunus dulcis* + *Pinus sylvestris*	1.42%	4.85%	3.41
Cisplatin	0.74 mM	1.21 mM	1.63

## Data Availability

The data supporting the findings of this study are contained within the article and its Supplementary Evaluative Materials, where applicable. Additional raw data may be made available by the corresponding author upon reasonable request.

## Supplementary Materials

The following supporting information can be downloaded at: https://www.mdpi.com/article/10.3390/life16081314/s1, Table S1. GC-MS-guided comparator-based docking scores (kcal/mol) of representative formulation-associated phytochemical markers across the melanoma-relevant target panel. Table S2. GC-MS-guided within-target ligand ranking and score differences relative to the corresponding comparator used in the screening workflow. Numerical coefficients are retained from the docking matrix, while ligand labels are aligned to the chemically identified formulation markers. Table S3. Pose-level internal descriptors for top-ranked GC-MS/FAME-aligned phytochemical markers. Table S4. Melanoma target ontology and rationale. Table S5. Reference inhibitors and binding context used for comparator-based docking in the transferred in silico framework. Table S6. α-Pinene and reference-drug docking scores across the melanoma target panel. Table S7. Viability of B16F10 melanoma cells and MRC-5 fibroblasts following treatment with different concentrations of cisplatin, as determined by the NRU assay. Data are presented as mean ± SE with *p* values. Table S8. Viability of B16F10 melanoma cells and MRC-5 fibroblasts following treatment with different concentrations of dual-oil phytochemical formulation “Nevus Recovery”: *P. dulcis* + *P. sylvestris*, as determined by the NRU assay. Data are presented as mean ± SE with p values. Table S9. IC50 and SI values of investigated treatments. Table S10. Summary of maximal inhibiting concentration annotations for the tested preparations. The table is reproduced as a descriptive record of the provided experimental interpretation and does not replace fitted IC50 estimation or formal dose-response modeling. Table S11. Quantitative and relative composition of the main fatty acids in almond oil, determined as fatty acid methyl esters. Table S12. Complete fatty acid profile of almond oil determined by GC/MS analysis of fatty acid methyl esters. Table S13. Major constituents and grouped chemical composition of pine needle essential oil determined by GC/MS. Table S14. Complete chemical composition of pine needle essential oil determined by GC/MS analysis. Table S15. Recent 2025 literature comparators supporting the GC-MS/FAME interpretation of the *Prunus dulcis* and *Pinus sylvestris* oil profiles used in this work.

## Abbreviations

4PL	Four-parameter logistic model
B16F10	Murine malignant melanoma cell line
BRAF V600E	BRAF valine-to-glutamate substitution at codon 600
CDK4/6	Cyclin-dependent kinases 4 and 6
COX-2	Cyclooxygenase-2
ERK2	Extracellular signal-regulated kinase 2
IFP	Interaction fingerprint
KIT	KIT proto-oncogene receptor tyrosine kinase
MAPK	Mitogen-activated protein kinase pathway
MEK1	Mitogen-activated protein kinase 1
PARP1	Poly(ADP-ribose) polymerase 1
RMSD	Root-mean-square deviation
TYR	Tyrosinase
FAME	Fatty acid methyl ester
FID	Flame ionization detector
GC/MS	Gas chromatography/mass spectrometry
IC_50_	Half-maximal inhibitory concentration
MRC-5	Human fibroblast cell line
NRU	Neutral-red uptake
ROS	Reactive oxygen species
SI	Selectivity index
*v/v*	Volume/volume
