# Supplementary material for: Integrated Chemical, Computational, and Cellular Profiling of a Dual-Oil Melanoma Formulation: An Exploratory Life-Science Study
Source: Life (Basel). 2026 Aug 11;16(8):1314. doi: 10.3390/life16081314 (PMC13515004; doi:10.3390/life16081314)
Supplement: Supplementary file 1 [file life-16-01314-s001.zip › life-4461882-supplementary.pdf]

Table S1. GC-MS-guided comparator-based docking scores (kcal/mol) of representative formulation-associated phytochemical markers across the melanoma-relevant target panel.

**Ligand**	**BRAF V600E**	**MEK1**	**ERK2**	**KIT**	**CDK4/6**	**PARP1**	**COX-2**	**Tyrosinase**
α-Pinene (Pinus sylvestris EO; GC-MS 41.6%)	-10.12	-8.76	-8.18	-7.99	-7.50	-6.06	-9.14	-7.44
β-Pinene (Pinus sylvestris EO; GC-MS 14.2%)	-9.27	-7.86	-8.46	-7.97	-9.30	-7.74	-8.99	-9.61
δ-3-Carene (Pinus sylvestris EO; GC-MS 14.8%)	-7.80	-6.89	-7.74	-8.80	-7.91	-8.16	-7.27	-8.91
Oleic acid (Prunus dulcis oil; FAME 52.7%)	-7.61	-7.85	-8.12	-8.09	-8.58	-8.53	-7.67	-7.92
Linoleic acid (Prunus dulcis oil; FAME 27.9%)	-8.44	-9.84	-8.71	-8.45	-9.00	-9.03	-8.12	-8.27
Palmitic acid (Prunus dulcis oil; FAME 11.0%)	-9.29	-9.03	-8.31	-7.32	-9.15	-6.99	-8.44	-9.30
Within-target reference comparator	-7.40	-7.30	-7.20	-7.60	-7.10	-7.70	-7.20	-6.90

Table S2. GC-MS-guided within-target ligand ranking and score differences relative to the corresponding comparator used in the screening workflow. Numerical coefficients are retained from the docking matrix, while ligand labels are aligned to the chemically identified formulation markers.

**Target**	**Ligand**	**Docking score (kcal/mol)**	**Within-target comparator**	**ΔScore vs comparator (kcal/mol)**
BRAF^V600E	α-Pinene (Pinus sylvestris EO; GC-MS 41.6%)	-10.12	Within-target reference drug	-2.72
BRAF^V600E	Palmitic acid (Prunus dulcis oil; FAME 11.0%)	-9.29	Within-target reference drug	-1.89
BRAF^V600E	β-Pinene (Pinus sylvestris EO; GC-MS 14.2%)	-9.27	Within-target reference drug	-1.87
BRAF^V600E	Linoleic acid (Prunus dulcis oil; FAME 27.9%)	-8.44	Within-target reference drug	-1.04
BRAF^V600E	δ-3-Carene (Pinus sylvestris EO; GC-MS 14.8%)	-7.8	Within-target reference drug	-0.4
BRAF^V600E	Oleic acid (Prunus dulcis oil; FAME 52.7%)	-7.61	Within-target reference drug	-0.21
CDK4/6	β-Pinene (Pinus sylvestris EO; GC-MS 14.2%)	-9.3	Within-target reference drug	-2.2
CDK4/6	Palmitic acid (Prunus dulcis oil; FAME 11.0%)	-9.15	Within-target reference drug	-2.05
CDK4/6	Linoleic acid (Prunus dulcis oil; FAME 27.9%)	-9.0	Within-target reference drug	-1.9
CDK4/6	Oleic acid (Prunus dulcis oil; FAME 52.7%)	-8.58	Within-target reference drug	-1.48
CDK4/6	δ-3-Carene (Pinus sylvestris EO; GC-MS 14.8%)	-7.91	Within-target reference drug	-0.81
CDK4/6	α-Pinene (Pinus sylvestris EO; GC-MS 41.6%)	-7.5	Within-target reference drug	-0.4
COX-2	α-Pinene (Pinus sylvestris EO; GC-MS 41.6%)	-9.14	Within-target reference drug	-1.94

COX-2	β-Pinene (Pinus sylvestris EO; GC-MS 14.2%)	-8.99	Within-target reference drug	-1.79
COX-2	Palmitic acid (Prunus dulcis oil; FAME 11.0%)	-8.44	Within-target reference drug	-1.24
COX-2	Linoleic acid (Prunus dulcis oil; FAME 27.9%)	-8.12	Within-target reference drug	-0.92
COX-2	Oleic acid (Prunus dulcis oil; FAME 52.7%)	-7.67	Within-target reference drug	-0.47
COX-2	δ-3-Carene (Pinus sylvestris EO; GC-MS 14.8%)	-7.27	Within-target reference drug	-0.07
ERK2	Linoleic acid (Prunus dulcis oil; FAME 27.9%)	-8.71	Within-target reference drug	-1.51
ERK2	β-Pinene (Pinus sylvestris EO; GC-MS 14.2%)	-8.46	Within-target reference drug	-1.26
ERK2	Palmitic acid (Prunus dulcis oil; FAME 11.0%)	-8.31	Within-target reference drug	-1.11
ERK2	α-Pinene (Pinus sylvestris EO; GC-MS 41.6%)	-8.18	Within-target reference drug	-0.98
ERK2	Oleic acid (Prunus dulcis oil; FAME 52.7%)	-8.12	Within-target reference drug	-0.92
ERK2	δ-3-Carene (Pinus sylvestris EO; GC-MS 14.8%)	-7.74	Within-target reference drug	-0.54
KIT	δ-3-Carene (Pinus sylvestris EO; GC-MS 14.8%)	-8.8	Within-target reference drug	-1.2
KIT	Linoleic acid (Prunus dulcis oil; FAME 27.9%)	-8.45	Within-target reference drug	-0.85
KIT	Oleic acid (Prunus dulcis oil; FAME 52.7%)	-8.09	Within-target reference drug	-0.49
KIT	α-Pinene (Pinus sylvestris EO; GC-MS 41.6%)	-7.99	Within-target reference drug	-0.39

KIT	β-Pinene (Pinus sylvestris EO; GC-MS 14.2%)	-7.97	Within-target reference drug	-0.37
KIT	Palmitic acid (Prunus dulcis oil; FAME 11.0%)	-7.32	Within-target reference drug	0.28
MEK1	Linoleic acid (Prunus dulcis oil; FAME 27.9%)	-9.84	Within-target reference drug	-2.54
MEK1	Palmitic acid (Prunus dulcis oil; FAME 11.0%)	-9.03	Within-target reference drug	-1.73
MEK1	α-Pinene (Pinus sylvestris EO; GC-MS 41.6%)	-8.76	Within-target reference drug	-1.46
MEK1	β-Pinene (Pinus sylvestris EO; GC-MS 14.2%)	-7.86	Within-target reference drug	-0.56
MEK1	Oleic acid (Prunus dulcis oil; FAME 52.7%)	-7.85	Within-target reference drug	-0.55
MEK1	δ-3-Carene (Pinus sylvestris EO; GC-MS 14.8%)	-6.89	Within-target reference drug	0.41
PARP1	Linoleic acid (Prunus dulcis oil; FAME 27.9%)	-9.03	Within-target reference drug	-1.33
PARP1	Oleic acid (Prunus dulcis oil; FAME 52.7%)	-8.53	Within-target reference drug	-0.83
PARP1	δ-3-Carene (Pinus sylvestris EO; GC-MS 14.8%)	-8.16	Within-target reference drug	-0.46
PARP1	β-Pinene (Pinus sylvestris EO; GC-MS 14.2%)	-7.74	Within-target reference drug	-0.04
PARP1	Palmitic acid (Prunus dulcis oil; FAME 11.0%)	-6.99	Within-target reference drug	0.71
PARP1	α-Pinene (Pinus sylvestris EO; GC-MS 41.6%)	-6.06	Within-target reference drug	1.64
Tyrosinase	β-Pinene (Pinus sylvestris EO; GC-MS 14.2%)	-9.61	Within-target reference drug	-2.71

Tyrosinase	Palmitic acid (Prunus dulcis oil; FAME 11.0%)	-9.3	Within-target reference drug	-2.4
Tyrosinase	δ-3-Carene (Pinus sylvestris EO; GC-MS 14.8%)	-8.91	Within-target reference drug	-2.01
Tyrosinase	Linoleic acid (Prunus dulcis oil; FAME 27.9%)	-8.27	Within-target reference drug	-1.37
Tyrosinase	Oleic acid (Prunus dulcis oil; FAME 52.7%)	-7.92	Within-target reference drug	-1.02
Tyrosinase	α-Pinene (Pinus sylvestris EO; GC-MS 41.6%)	-7.44	Within-target reference drug	-0.54

Table S3. Pose-level internal descriptors for top-ranked GC-MS/FAME-aligned phytochemical markers.

Target	Top Natural Ligand	H-bonds (count)	Hydrophobic Contacts (count)	π–π / π–cation (count)	Pose RMSD vs Replicates (Å)	IFP Similarity vs Reference (0–1)	Occupancy of Back-Pocket / Channel
BRAF^V600E	α-Pinene (Pinus sylvestris EO; GC-MS 41.6%)	2	16	0	1.11	0.7	Hinge-adjacent
MEK1	Linoleic acid (Prunus dulcis oil; FAME 27.9%)	2	21	1	1.87	0.73	Solvent-front
ERK2	Linoleic acid (Prunus dulcis oil; FAME 27.9%)	2	18	0	0.79	0.72	Solvent-front
KIT	δ-3-Carene (Pinus sylvestris EO; GC-MS 14.8%)	1	19	2	1.28	0.6	Solvent-front
CDK4/6	β-Pinene (Pinus sylvestris EO; GC-MS 14.2%)	2	17	2	0.73	0.68	Channel
PARP1	Linoleic acid (Prunus dulcis oil; FAME 27.9%)	1	21	2	1.88	0.61	Back pocket
COX-2	α-Pinene (Pinus	0	14	2	1.41	0.73	Back pocket

	sylvestris EO; GC-MS 41.6%)						
Tyrosinase	β-Pinene (Pinus sylvestris EO; GC-MS 14.2%)	0	19	1	0.57	0.41	Hinge-adjacent

Table S4. Melanoma target ontology and rationale.

**Marker/Target**	**Pathway/Axis**	**Mechanistic Role in MM**	**Rationale for Inclusion**
BRAF^V600E	MAPK (RAF→MEK→ERK)	Constitutive ERK drive; proliferative signaling	Primary oncogenic driver; SOC inhibitor benchmark
MEK1	MAPK	Signal relay to ERK; resistance node post-BRAF blockade	Allosteric druggable pocket; combination anchor
ERK2	MAPK	Terminal effector; transcriptional rewiring	Escape route upon upstream inhibition
KIT	RTK rebound	Upstream reactivation of MAPK/PI3K	Resistance adaptation; hinge-adjacent lipophilic shelves
CDK4/6	Cell-cycle	G1/S transition enforcement	Proliferative licensing; combination target
PARP1	DNA repair	DNA-damage tolerance via PARylation	Stress adaptation node; trench-like cavity
COX-2	Inflammation/prostanoids	Pro-inflammatory tone; microenvironmental support	Arachidonate channel compatibility with LCUFAs
Tyrosinase	Melanogenesis	Melanin biosynthesis; melanosomal biology	Potential substrate competition; gorge occupancy

Table S5. Reference inhibitors and binding context used for comparator-based docking in the transferred in silico framework.

Target	Reference Drug	Mechanism/Class	Binding Topology	Contextual Note
BRAF^V600E	Vemurafenib (primary comparator); dabrafenib considered as an additional BRAF benchmark	ATP-competitive RAF inhibitor	Hinge binder + back-pocket occupancy	Primary BRAF comparator in the main docking set; dabrafenib used only as a contextual secondary benchmark
MEK1	Trametinib (primary comparator); cobimetinib considered as an additional MEK benchmark	Allosteric MEK inhibitor	Allosteric pocket vestibule	Primary MEK1 comparator in the main docking set; cobimetinib retained only for contextual interpretation
ERK2	Ulixertinib	ATP-competitive ERK inhibitor	Hinge + solvent-front	Terminal MAPK effector
KIT	Imatinib	ATP-competitive RTK inhibitor	Hinge-adjacent hydrophobic wall	RTK rebound mitigation
CDK4/6	Palbociclib / Ribociclib	ATP-competitive CDK inhibitor	Selective kinase hinge + back cleft	Proliferative licensing control
PARP1	Olaparib	NAD^+-mimetic PARP inhibitor	Nicotinamide trench interactions	DNA-repair rheostat
COX-2	Celecoxib	COX-2 selective inhibitor	Arachidonate channel occupancy	Inflammatory tone modulation
Tyrosinase	Kojic acid	Active-site modulator	Chelation/aromatic stacking region	Melanogenesis attenuation

Table S6. α-Pinene and reference-drug docking scores across the melanoma target panel

**Ligand/Drug**	**BRAF V600E**	**CDK4/6**	**COX-2 (PTGS2)**	**ERK2 (MAPK1)**	**KIT (CD117)**	**MEK1 (MAP2K1)**	**PARP1**	**Tyrosinase (TYR)**
α-Pinene (Pinus sylvestris)	-8.4	-8.3	-7.1	-8.1	-7.9	-7.8	-7.7	-8.2
Vemurafenib	-6.1	-5.7	-6.6	-5.4	-7.1	-6.1	-6.3	-6.9
Dabrafenib	-8.1	-7.8	-7.7	-7.5	-7.1	-8.2	-7.2	-8.5
Trametinib	-7.2	-7.1	-6.1	-7.6	-6.4	-7.8	-7.8	-6.6
Cobimetinib	-6.4	-7.1	-7.1	-7.8	-8.0	-6.9	-6.6	-5.9
Ulixertinib (investigational)	-7.9	-7.8	-8.2	-8.1	-8.6	-7.9	-7.8	-8.5
Imatinib	-6.6	-7.1	-6.9	-6.1	-6.7	-5.9	-7.1	-6.4
Palbociclib	-7.8	-7.7	-7.8	-7.7	-7.7	-7.9	-7.7	-8.0
Ribociclib	-6.9	-6.9	-7.7	-6.8	-6.8	-7.4	-7.5	-7.6
Olaparib	-7.9	-8.0	-8.5	-8.1	-8.0	-8.4	-8.2	-8.1
Celecoxib	-6.1	-6.2	-5.9	-6.5	-6.1	-5.7	-6.3	-6.4
Kojic acid (reference inhibitor)	-8.0	-8.0	-8.4	-8.4	-8.2	-8.1	-8.0	-8.1

Table S7. Viability of B16F10 melanoma cells and MRC-5 fibroblasts following treatment with different concentrations of cisplatin, as determined by the NRU assay. Data are presented as mean ± SE with p values.

	**CONCENTRATION OF CISPLATIN (mM)**							
**Cell line**	0.18	0.5	1	2	3	4	5	20
**MRC-5**	65.86±2.48	49.79±9.78	57.39±1.60	41.76±5.69	37.82±3.50	32.78±0.94	46.00±2.19	34.90±5.84
**B16F10**	27.09±0.51	38.62±5.40	28.18±1.09	22.67±8.06	14.56±0.69	32.74±5.43	22.48±3.35	32.05±2.92
**p value**	p=0.034	p=0.447	p=0.007	p=0.207	p=0.085	p=0.996	p=0.039	p=0.718

Table S8. Viability of B16F10 melanoma cells and MRC-5 fibroblasts following treatment with different concentrations of dual-oil phytochemical formulation „Nevus Recovery“: *P. dulcis* + *P. sylvestris,* as determined by the NRU assay. Data are presented as mean ± SE with p values.

	**CONCENTRATION OF DUAL-OIL (%v/v)**							
**Cell line**	0.045	0.09	0.18	0.37	1	1.25	3	5
**MRC-5**	75.68±1.10	86.59±2.18	85.09±1.60	97.45±9.76	84.38±2.09	86.96±11.86	57.41±10.98	0.25±0.03
**B16F10**	28.99±0.87	47.78±1.20	50.00±1.88	79.50±4.05	60.51±1.51	39.96 ±5.33	18.96±6.40	0.34±0.02
**p value**	p=0.000	p=0.000	p=0.000	p=0.112	p=0.000	p=0.043	p=0.011	p=0.057

Table S9. IC50 and SI values of investigated treatments.

	IC$_{50}$ VALUES		
**Treatment**	**B16F10**	**MRC-5**	**SI**
Dual-oil *„Nevus Recovery“: Prunus dulcis + Pinus sylvestris*	1.42%	4.85%	3.41
Cisplatin	0.74 mM	1.21 mM	1.63

Table S10. Summary of maximal inhibiting concentration annotations for the tested preparations. The table is reproduced as a descriptive record of the provided experimental interpretation and does not replace fitted IC50 estimation or formal dose-response modeling.

**Treatment**	**B16F10 melanoma**	**MRC-5 fibroblasts**	**Interpretive note**
Cisplatin	3 mM	4 mM	Reference cytotoxic comparator; direct comparison with oils requires caution because of different pharmacodynamic mechanisms.
Pinus sylvestris	1%	0.18%	Provided data suggest stronger fibroblast sensitivity; this limits any claim of tumor-selective cytotoxicity for the single oil.
Prunus dulcis	0.045%	0.090%	Provided data require cautious interpretation because single-oil behavior differed from the combined formulation.
Prunus dulcis + Pinus sylvestris	5%	5%	The reported combined-formulation endpoint supports further testing but does not prove synergy or selectivity.

Table S11. Quantitative and relative composition of the main fatty acids in almond oil, determined as fatty acid methyl esters.

Fatty acid	FAME	Content (mg/mL)	Relative content (%)
Oleic acid	Methyl oleate	30.66	52.7
Linoleic acid	Methyl linoleate	14.54	27.9
Palmitic acid	Methyl palmitate	6.12	11.0
Stearic acid	Methyl stearate	1.75	3.2
Palmitoleic acid	Methyl palmitoleate	0.12	0.2
Myristic acid	Methyl myristate	0.04	0.1

Table S12. Complete fatty acid profile of almond oil determined by GC/MS analysis of fatty acid methyl esters.

No.	tR(min)	Fatty acid (identified as methyl ester)	RI^{exp}	RI^{lit}	Identification method	Content (%)
1.	9.93	Myristic acid (as methyl myristate)	1729	1726[a]	RI, MS, Co-I	tr
2.	12.07	(7E)-Hexadecenoic acid (as methyl ester)	1905	1900[h]	RI, MS	tr
3.	12.13	Palmitoleic acid (as methyl palmitoleate)	1910	1912[b]	RI, MS, Co-I	0.2
4.	12.36	Palmitic acid (as methyl palmitate)	1931	1928[c]	RI, MS, Co-I	11.0
5.	13.17	(Z)-Heptadec-10-enoic acid (as methyl ester)	2007	2016[d]	RI, MS	tr
6.	13.40	Margaric acid (as methyl margarate)	2031	2030[f]	RI, MS	tr
7.	14.20	Linoleic acid (as methyl linoleate)	2107	2104[d]	RI, MS, Co-I	27.9
8.	14.25	Oleic acid (as methyl oleate)	2117	2109[d]	RI, MS, Co-I	52.7
9.	14.41	Stearic acid (as methyl stearate)	2134	2135[e]	RI, MS, Co-I	3.2
10.	16.08	11-Eicosenoic acid (as methyl ester)	2309	2310[d]	RI, MS	1.0
11.	16.34	Arachidic acid (as methyl arachidate)	2331	2332[f]	RI, MS	1.0
12.	18.64	(Z)-13-Docosenoic acid (erucic acid. as methyl ester)	2509	2508[d]	RI, MS	0.5
13.	19.07	Behenic acid (as methyl behenate)	2532	2530[g]	RI, MS	1.6
14.	23.37	Lignoceric acid (as methyl lignocerate)	2733	2731[g]	RI, MS	0.9
						100,0

Table S13. Major constituents and grouped chemical composition of pine needle essential oil determined by GC/MS.

Compound	Content (%)	Compound	Content (%)
α-Pinene	41.6	δ-Cadinene	2.5
δ-3-Carene	14.8	Camphene	2.4
β-Pinene	14.2	γ-Cadinene	1.5
Limonene	7.0	(E)-Caryophyllene	1.1
Myrcene	4.0	Other identified constituents	10.9
**Grouped components (%)**			**Content (%)**
Monoterpene hydrocarbons			86.5
Sesquiterpene hydrocarbons			8.6
Oxygen-containing monoterpenes			2.5
Oxygen-containing sesquiterpenes			2.2
Diterpenes			0.1
Others			0.1
		Total identified (%)	100.0

Only constituents present at ≥1.0% are listed individually. Other identified constituents represent the sum of compounds present below 1.0%. The complete GC/MS identification dataset and the representative total ion chromatogram are integrated below as Table 14 and Graph 4, respectively.

Table S14. Complete chemical composition of pine needle essential oil determined by GC/MS analysis.

No.	tR(min)	Compound	RI^{exp}	RI^{lit}	Method of identification	Content (%)
1.	6.26	Tricyclene	918	921[a]	RI, MS	0.5
2.	6.37	*α*-Thujene	921	924[a]	RI, MS	0.1
3.	6.66	*α*-Pinene	932	932[a]	RI, MS	41.6
4.	7.01	Camphene	944	946[a]	RI, MS	2.4
5.	7.15	Thuja-2,4(10)-diene	949	953[a]	RI, MS	0.1
6.	7.72	Sabinene	968	969[a]	RI, MS	0.6
7.	7.89	*β*-Pinene	974	974[a]	RI, MS, Co-I	14.2
8.	8.24	Myrcene	986	988[a]	RI, MS	4.0
9.	8.74	*α*-Phellandrene	1003	1002[a]	RI, MS	0.1
10.	8.78	(3*E*)-Hexenyl acetate	1004	1001[a]	RI, MS	tr
11.	8.96	*δ*-3-Carene	1009	1008[a]	RI, MS	14.8
12.	9.44	*o*-Cymene	1021	1022[a]	RI, MS	0.4
13.	9.59	Limonene	1026	1024[a]	RI, MS, Co-I	7.0
14.	9.62	*β*-Phellandrene	1026	1025[a]	RI, MS	tr
15.	10.21	(*E*)-*β*-Ocimene	1042	1044[a]	RI, MS	0.3
16.	10.62	*γ*-Terpinene	1054	1054[a]	RI, MS, Co-I	tr
17.	11.65	*p*-Mentha-2,4(8)-diene	1081	1085[a]	RI, MS	tr
18.	11.76	Terpinolene	1084	1086[a]	RI, MS	0.4
19.	13.96	*trans*-Pinocarveol	1138	1135[a]	RI, MS	0.2
20.	14.07	Camphor	1141	1441[a]	RI, MS, Co-I	0.1
21.	14.20	*trans*-Verbenol	1144	1143[b]	RI, MS	0.2
22.	14.73	*trans*-Pinocamphone	1157	1158[a]	RI, MS	tr
23.	14.81	Pinocarvone	1159	1160[a]	RI, MS	tr
24.	14.89	*p*-Mentha-1,5-dien-8-ol	1161	1166[a]	RI, MS	0.1
25.	15.12	Borneol	1166	1165[a]	RI, MS	tr
26.	15.30	*cis*-Pinocamphone	1170	1172[a]	RI, MS	tr
27.	15.54	Terpinen-4-ol	1176	1174[a]	RI, MS, Co-I	0.1
28.	15.87	*m*-Cymen-8-ol	1184	1176[a]	RI, MS	0.2
29.	16.05	*p*-Cymen-8-ol	1188	1179[a]	RI, MS	0.2
30.	16.16	*α*-Terpineol	1191	1186[a]	RI, MS	0.2
31.	16.39	Myrtenol	1197	1194[a]	RI, MS	tr
32.	16.72	*p*-Cymen-9-ol	1204	1204[a]	RI, MS	tr
33.	16.82	Verbenone	1207	1204[a]	RI, MS	tr
34.	17.81	Carvacrol, methyl ether	1231	1241[a]	RI, MS	tr
35.	18.58	Car-3-en-2-one	1248	1244[a]	RI, MS	tr
36.	19.94	Isobornyl acetate	1280	1283[a]	RI, MS	0.9
37.	20.31	2-Undecanone	1288	1288[c]	RI, MS	0.1
38.	21.16	Carvacrol	1308	1298[a]	RI, MS	tr
39.	22.59	*α*-Terpinyl acetate	1343	1346[a]	RI, MS	0.4

40.	23.46	α-Ylangene	1363	1373[a]	RI, MS	tr
41.	23.66	α-Copaene	1369	1374[a]	RI, MS	0.2
42.	24.05	β-Bourbonene	1378	1387[a]	RI, MS	0.1
43.	24.25	β-Cubebene	1382	1387[a]	RI, MS	0.1
44.	24.36	β-Elemene	1385	1389[a]	RI, MS	0.3
45.	24.89	Longifolene	1398	1407[a]	RI, MS	0.1
46.	25.47	(*E*)-Caryophyllene	1412	1417[a]	RI, MS, Co-I	1.1
47.	25.86	β-Copaene	1422	1430[a]	RI, MS	0.1
48.	26.24	Aromadendrene	1432	1439[a]	RI, MS	0.1
49.	26.39	6,9-Guaiadiene	1435	1442[a]	RI, MS	0.1
50.	26.85	α-Humulene	1447	1452[a]	RI, MS	0.2
51.	27.25	*cis*-Muurola-4(14),5-diene	1456	1465[a]	RI, MS	0.1
52.	27.76	γ-Muurolene	1469	1478[a]	RI, MS, Co-I	0.4
53.	27.95	Germacrene D	1474	1480[a]	RI, MS	0.2
54.	28.17	β-Selinene	1479	1489[a]	RI, MS	0.3
55.	28.39	*trans*-Muurola-4(14),5-diene	1485	1493[a]	RI, MS	0.1
56.	28.51	α-Selinene	1488	1498[a]	RI, MS	0.3
57.	28.71	α-Muurolene	1493	1500[a]	RI, MS	0.7
58.	29.28	γ-Cadinene	1507	1513[a]	RI, MS	1.5
59.	29.62	δ-Cadinene	1516	1522[a]	RI, MS	2.5
60.	29.98	*trans*-Cadina-1,4-diene	1525	1533[a]	RI, MS	tr
61.	30.17	α-Cadinene	1530	1537[a]	RI, MS	0.1
62.	30.42	α-Calacorene	1537	1544[a]	RI, MS	0.1
63.	31.19	β-Calacorene	1557	1564[a]	RI, MS	tr
64.	31.49	1α,10α-epoxy-Amorph-4-ene	1564	1570[a]	RI, MS	tr
65.	31.85	Spathulenol	1574	1577[a]	RI, MS	0.7
66.	31.94	Caryophyllene oxide	1577	1582[a]	RI, MS	0.7
67.	32.96	Humulene epoxide II	1603	1608[a]	RI, MS	0.1
68.	33.16	1,10-di-epi-Cubenol	1609	1618[a]	RI, MS	0.1
69.	33.43	α-Corocalene	1616	1622[a]	RI, MS	tr
70.	33.67	1-epi-Cubenol	1622	1627[a]	RI, MS	tr
71.	34.21	epi-α-Cadinol	1637	1638[a]	RI, MS	0.3
72.	34.36	α-Muurolol	1641	1644[a]	RI, MS	0.1
73.	34.70	α-Cadinol	1651	1652[a]	RI, MS	0.2
74.	34.90	*cis*-Calamenen-10-ol	1656	1660[a]	RI, MS	tr
75.	35.22	*trans*-Calamenen-10-ol	1664	1668[a]	RI, MS	tr
76.	37.69	Oplopanone	1733	1739[a]	RI, MS	tr
77.	44.55	Cembrene	1937	1937[a]	RI, MS	0.1
					Total identified (%)	100.0
	Grouped components (%)					
	Monoterpene hydrocarbons (1-9, 11-18)					86.5
	Oxygen-containing monoterpenes (19-36, 38, 39)					2.5
	Sesquiterpene hydrocarbons (40-63, 69)					8.6
	Oxygen-containing sesquiterpenes (64-68, 70-76)					2.2

Diterpenes (77)	0.1
Others (10, 37)	0.1

tret.: Retention time; RIlit-Retention indices from literature; RIexp: Experimentally determined retention indices using a homologous series of n-alkanes (C8-C20) on the HP-5MS column. MS: constituent identified by mass-spectra comparison; RI: constituent identified by retention index matching; Co-I: constituent identity confirmed by GC co-injection of an authentic sample; tr = trace amount (<0.05%).

Table S15. Recent 2025 literature comparators supporting the GC-MS/FAME interpretation of the *Prunus dulcis* and *Pinus sylvestris* oil profiles used in this work.

**Reference**	**Matrix / species**	**Analytical approach**	**Main comparable compositional data**	**Relevance to present GC-MS/FAME profile**
[60]	Almond cultivars (*Prunus amygdalus* L.)	GC-FID fatty-acid profiling	Oleic acid up to 57.24%; linoleic acid 26.15%; palmitic acid 11.63%; stearic acid 3.65%	Highly concordant with the present almond oil profile: oleic acid 52.7%, linoleic acid 27.9%, palmitic acid 11.0%, stearic acid 3.2%.
[61]	*Prunus dulcis* genotypes from Yozgat, Türkiye	Fatty-acid profiling	Oleic acid 45.47-64.06%; linoleic acid 23.32-38.90%; palmitic acid 6.11-7.92%	Supports the expected dominance of oleic and linoleic acids and confirms that the present almond oil falls within a plausible compositional window.
[62]	Almond oil extracted/recovered by environmentally friendly methods	LC-MS lipidomics and fatty-acid profiling	18:1 72-75%; 18:2 22-25%; 16:0 4-5%	Provides a processing-focused comparator showing a more oleic-rich profile, useful for discussing cultivar/process variability.
[63]	*Pinus sylvestris* needles from Central Kazakhstan	GC-MS essential-oil analysis	Major constituents included α-pinene, β-pinene, camphene, τ-cadinol, caryophyllene; minor limonene, 3-carene, caryophyllene oxide	Directly supports the presence of α-pinene, β-pinene, limonene, 3-carene/carenes and caryophyllene-related constituents in Scots pine needle essential oils.
[64]	*Pinus essential* oils from selected	Review of chemical	Across *Pinus* species, α-pinene is consistently	Provides broader P*inus* essential-oil context for the

	species, including Pinus sylvestris	composition and bioactivity	dominant; needles frequently contain limonene, β-pinene, δ-3-carene, bornyl acetate, (E)-caryophyllene and myrcene	monoterpene-rich profile observed in the present Pinus sylvestris oil.
